# The miR-302 cluster-IRFs-IRF1AS axis regulates influenza A virus replication in a species-specific manner

**DOI:** 10.1128/mbio.01375-25

**Published:** 2025-07-08

**Authors:** Lingcai Zhao, Chenglin Hou, Xifeng Hu, Chenfeng Jiang, Shengmin Li, Jun Xia, Jihui Ping

**Affiliations:** 1 MOE Joint International Research Laboratory of Animal Health and Food Safety, Engineering Laboratory of Animal Immunity of Jiangsu Province, College of Veterinary Medicine, Nanjing Agricultural University 261674, Nanjing, China; 2 Xinjiang Academy of Animal Sciences, Institute of Veterinary Medicine (Research Center of Animal Clinical), Urumqi, China; Huazhong Agricultural University, Wuhan, Hubei, China

**Keywords:** influenza A virus, miR-302 cluster-IRFs, IRFs-IRF1AS, enhancer cluster, enhancer RNAs

## Abstract

Non-coding RNAs are crucial orchestrators in the intricate dance between viruses and host cells, among which the expression and function of enhancer RNAs (eRNAs) during influenza virus infection remain largely unexplored. This study utilized whole transcriptome high-throughput sequencing to investigate the molecular mechanisms underpinning the species-specific regulation of influenza virus replication by the miR-302 cluster-IRFs-IRF1AS axis both *in vivo* and *in vitro*. Mechanistically, the CTNNB1-induced miR-302 cluster targeted various interferon regulatory factors (mainly IRF1 and IRF2) with varying affinities and silencing efficiencies, except for miR-302e and miR-302f. Furthermore, miR-302 cluster-IRFs triggered the induction of interferon-induced hub genes and hub lncRNAs defined through weighted gene co-expression network analysis. Importantly, the intricate interplay between IRFs, direct targets of the miR-302 cluster, and IRF1AS, an indirect target, in terms of gene loci and transcriptional regulation reveals a crosstalk in the miR-302 cluster-IRFs-IRF1AS axis. That is, on the one hand, IRF1 and IRF7 bind to the promoter of IRF1AS to promote the transcription of eRNAs. On the other hand, IRF1AS functions as an enhancer cluster that orchestrates and *cis*-regulates the transcription of IRF1, thereby rapidly amplifying the antiviral immune response initiated by miR-302 cluster-IRFs. In conclusion, we have unveiled a novel regulatory network governed by the miR-302 cluster-IRFs-IRF1AS, offering fresh perspectives on immune regulatory mechanisms.

**IMPORTANCE:**

Non-coding RNAs play a crucial role in regulating the three-dimensional structure of chromatin. They influence gene expression through various mechanisms and thereby contribute to the onset and progression of influenza A virus pathogenicity. Our comprehensive whole transcriptome sequencing analysis reveals a novel finding: the species-specific regulation of influenza virus replication by the miR-302 cluster-IRFs-IRF1AS axis. Our findings indicate that the miR-302 cluster-IRFs axis facilitates the transcription of key hub genes and hub lncRNAs, most of which significantly inhibit influenza virus replication. Notably, the downstream IRF1AS assembles into an enhancer cluster, orchestrating and *cis*-regulating the transcription of IRF1 to activate the interferon system. This investigation enhances our understanding of the regulatory network underlying viral infections and offers novel insights into immune regulatory mechanisms.

## INTRODUCTION

Influenza A virus (IAV), a member of the influenza virus family, is widely recognized as a highly destructive pathogen that poses a grave threat to both human and animal populations. It is known for its extensive global spread and severe impact on health (1–4). Over the past century, humanity has witnessed four influenza pandemics, resulting in a tragic loss of millions of lives. IAV is a seasonal infection that recurs annually, with the emergence of new mutant strains capable of triggering pandemics (5, 6). Understanding how IAV interacts with host factors and uncovering the underlying mechanisms is essential for effective influenza prevention and control.

IAV is an enveloped, segmented, single-stranded, negative-sense RNA virus with a genome length of approximately 13.6 kb, comprised eight gene segments (7). Following viral entry into host cells, the innate immune response is initiated by activating pattern recognition receptors (PRRs) present in immune cells, notably macrophages and neutrophils. Several PRRs detect viral infections, including membrane-bound Toll-like receptors, and cytosolic receptors such as cGAS and RIG-I-like receptors (RLRs) (8). The negative-sense RNA genome of IAV is recognized by RLRs, primarily retinoic acid-inducible gene-I (RIG-I/DDX58). After recognizing viral RNA, RIG-I undergoes conformational changes and interacts with mitochondrial antiviral-signaling protein (MAVS), which then activates TBK1/IKKε and TAK1/IKK kinases and subsequent phosphorylation of IRF3 and NF-κB (9). Interferon (IFN) acts through its cognate receptors (IFNAR1/2 or IFNLR/IL10R2), leading to the formation of the transcriptionally active STAT1 and STAT2, and ultimately activating the transcription of scads of inflammatory and antiviral target genes (10, 11).

Most of the human genome is transcribed into non-coding RNAs (ncRNAs), which are not translated into proteins (12). ncRNAs are classified by length into several types, including microRNAs (~22 nt), siRNAs (21–25 nt), piRNAs (24–33 nt), vault RNAs (80–150 nt), long non-coding RNAs (>200 nt), and some circular RNAs derived from lncRNAs. These ncRNAs serve as critical regulatory elements in diverse cellular processes, encompassing cell growth, differentiation, survival, and apoptosis (13–15). Notably, research has highlighted the role of circVAMP3 in functioning as a decoy for viral NP and NS1 proteins, disrupting their functions (16). AIVR sequesters miRNA, thereby promoting IFN production and limiting viral replication (17). On the other hand, some ncRNAs are hijacked by viruses for infection and replication. IFN-independent lncRNA-ACOD1 promotes viral replication by regulating cellular metabolism (18). IFN-induced long non-coding RNA lnc-Lsm3b competes with viral RNA for binding to RIG-I and interferes with cellular immune responses (19). In general, ncRNAs are key regulators of host-IAV interactions and play a crucial role in the pathogenesis of IAV (20). However, the regulatory mechanisms of most ncRNAs on influenza virus replication are still unclear.

Enhancers are regulatory DNA elements located between genes that control gene activation and repression in different combinations, at varying times and levels. Upon activation, enhancers induce local chromatin remodeling, exposing DNA motifs that recruit transcription factors, which in turn facilitate RNA polymerase binding. This process enables enhancers to transcribe non-coding RNAs, known as enhancer RNAs (eRNAs) (21, 22). Compared to other lncRNAs or mRNAs, eRNAs exhibit several distinct characteristics. They are bidirectionally transcribed from enhancer regions enriched with histone methylation marks, particularly H3K4me1 and H3K4me2 modifications (23). Unlike mRNAs, most eRNAs are shorter (~500 bp), non-polyadenylated, unstable, and predominantly localized in the nucleus (24). eRNAs play a crucial role in regulating chromatin three-dimensional structure and modulating gene expression through diverse mechanisms (25). However, studies on eRNAs in the context of influenza virus infection remain limited, and the functions and effects of most eRNAs are yet to be fully understood.

Presented here, our whole transcriptome sequencing analysis has unveiled a novel observation: the species-specific regulation of influenza virus replication by the miR-302 cluster-IRFs-IRF1AS axis *in vivo* and *in vitro*. Further analysis revealed that, with the exception of miR-302e and miR-302f, the CTNNB1-induced miR-302 cluster targeted various IFN regulatory factors with varying affinities and silencing efficiencies, activating the IFN-induced protein-coding genes and long non-coding RNAs. Interestingly, the IRF1AS acts as an enhancer cluster that coordinates and *cis*-regulates IRF1 transcription. In summary, this study proposed a novel miR-302 cluster-IRFs-IRF1AS axis, which shed light on immune regulatory mechanisms and laid the foundation for finding new antiviral targets and developing broad-spectrum anti-influenza virus drugs.

## MATERIALS AND METHODS

### Viruses and cells

HEK293T, A549, MDCK, DF-1, and PK-15 cells were cultured in Dulbecco’s modified Eagle’s medium (DMEM; Gibco, USA) and MLE12 in Dulbecco’s modified Eagle’s medium/Nutrient Mixture F-12 (DMEM/F12) supplemented with 10% fetal bovine serum (FBS; Gibco), 0.2% NaHCO_3_, 100 µg/mL streptomycin, and 100 IU/mL penicillin (Gibco) at 37°C with 5% CO_2_. IAV A/WSN/1933 (H1N1), CK/SH/49/19 (H9N2), and A/Hong Kong/01/1968 (H3N2) were generated by reverse genetics and inoculated into 10-day-old specific-pathogen-free chicken embryos for virus propagation (26).

### Plasmid construction

Cellular RNA was reverse transcribed into single-stranded DNA utilizing the RevertAid First Strand cDNA Synthesis Kit (K1622, Thermo Fisher Scientific, USA). Specific coding or non-coding gene sequences were amplified through PCR or overlapping PCR and subsequently subcloned into the pcDNA3.1(+) or pCAGGS vector, respectively. Genes of interest intended for overexpression in cell lines were cloned into the pMXs-IRES-Blasticidin retroviral vector. Retroviruses were produced in Plat-E cells, and cells infected with the retrovirus were screened using blasticidin-S (ST018, Beyotime, China) at a concentration of 10 µg/mL. Validation of all plasmids was carried out using Sanger sequencing. Details of primers used in this study are provided in Table S3.

### Western blot and antibodies

Briefly, the Western blotting assay was conducted following the previously established protocol (27). Antibodies used in this article are listed in Table S3.

### Isolation of nuclear and cytoplasmic proteins

Nuclear and cytoplasmic proteins were isolated separately using the Nuclear and Cytoplasmic Protein Extraction Kit (P0027, Beyotime) according to the manufacturer’s protocol.

### Isolation of nuclear and cytoplasmic RNAs

Nuclear and cytoplasmic RNAs were isolated separately using the Cytoplasmic and Nuclear RNA Purification Kit (AM1921, Thermo Fisher) according to the manufacturer’s protocol.

### siRNAs and miRNAs

MicroRNA mimics, siRNAs, and negative controls (NC siRNA or NC mimics) were obtained from GenePharma (Shanghai, China). For transfection, the cells were treated with 20 nM of microRNA mimics, siRNA, or negative control (NC siRNA or NC mimics) using Lipofectamine 2000 (Invitrogen, USA) in 24-well plates, following the manufacturer’s guidelines. The specific siRNAs and oligonucleotides used in this study are listed in Table S3.

### CRISPR-Cas9 system

Guide RNAs targeting specific genes were designed using an online tool (http://crispr.mit.edu), and the corresponding oligonucleotides were synthesized. The specific sgRNAs were then cloned into the BsmBI restriction site of the inducible lentiviral vector lentiGuide-Puro. To generate lentivirus, the lentiGuide-Puro plasmid was co-transfected into HEK293T cells along with the packaging plasmids pVSVg (AddGene 8454) and psPAX2 (AddGene 12260). Twelve hours post-transfection, the medium was replaced with fresh DMEM supplemented with 10% FBS. Viral supernatants were collected at 48 h, filtered through a 0.45 µm membrane, and used for infection. After lentiviral transduction, the cells were cultured for 48 h and then selected with puromycin (2 µg/mL). Polyclonal cell lines were validated by sequencing and immunoblotting and screened for mycoplasma contamination. The sgRNA sequences and corresponding sequencing results are detailed in Table S3.

### Quantitative real-time PCR (qPCR)

Cellular RNA extraction was performed according to a previously established protocol (28). To quantify mRNA or lncRNA, 1,000 ng of total RNA was subjected to first-strand cDNA synthesis using oligo(dT)_20_ or random hexamers with the HiScript II 1st Strand cDNA Synthesis Kit (R212, Vazyme, China). For miRNA quantification, total RNA was isolated using the MiPure Cell/Tissue miRNA Kit (RC201, Vazyme) to preserve small RNA following the manufacturer’s guidelines. Reverse transcription of miRNAs was carried out using custom-designed stem-loop reverse transcription primers. Subsequently, qPCR was performed using the synthesized cDNAs, gene-specific primer pairs, and AceQ qPCR SYBR Green Master Mix (Q111, Vazyme) on a Roche LightCycler 96. GAPDH served as the endogenous control for mRNA, while U6 was utilized for microRNAs. The 2^−ΔΔC*t*^ method was employed to determine the relative expression levels of the target genes. The primer sequences are detailed in Table S3.

### RNA immunoprecipitation (RIP)

A549 cells were seeded in 60 mm cell culture dishes and infected with WSN virus at a multiplicity of infection (MOI) of 1 for 12 h. Post-infection, the cells were harvested, lysed, and subjected to immunoprecipitation employing either anti-AGO2 (67934-1-Ig, Proteintech, China) or anti-IgG antibody. RNA extraction was carried out from the immunoprecipitated samples, and cDNA was synthesized using oligo(dT)_20_ for mRNA transcripts. The quantification of each transcript’s relative enrichment was conducted using quantitative real-time PCR. Initially, the relative enrichment values were normalized to the input and subsequently compared with data acquired from the immunoprecipitated sample for further analysis

### Chromatin immunoprecipitation (ChIP)-qPCR

Chromatin was isolated from A549 cells post-crosslinking with formaldehyde. Subsequently, DNA fragments of approximately 500 bp were generated using micrococcal nuclease (D7201S, Beyotime). The chromatin was then subjected to immunoprecipitation with specific antibodies and 50 µL of protein A/G slurry (sc-2003, Santa Cruz, USA), followed by overnight rotation at 4°C. After thorough washing with low salt, high salt, and TE buffer, the chromatin was treated with proteinase K and underwent reverse crosslinking at 65°C for 6 h. DNA purification was accomplished using the QIAquick PCR Purification Kit (Cat#28104, Qiagen). Target amplification and detection were carried out using qPCR. The results were analyzed utilizing the 2^−ΔΔC*t*^ method relative to the input DNA. The primer sequences are provided in Table S3.

### Luciferase reporter assay

To assess the activation of IFN-related pathways, HEK293T cells were co-transfected with IFN-β-luc, ISRE-luc, or NF-κB-luc reporter plasmids, along with the pRL-TK plasmid, specified gene expression plasmids, or an empty vector. After 24 hours of transfection, the cells were infected with Sendai virus to induce stimulation, and cell lysis was conducted 24 h post-infection.

For miRNA target gene analysis, predicted binding sites of the target genes or corresponding mutants were introduced into the pmir-GLO vector. Luciferase reporter vectors containing the WT 3′ UTR or MUT 3′ UTR of the gene, along with the pRL-TK plasmid, were co-transfected with NC mimics or designated miRNA mimics into HEK293T cells. Cell lysis was carried out 24 h after transfection.

To investigate gene promoter activity or enhancers, the gene promoter or genomic fragment was PCR-amplified and inserted into the pGL3-Basic vector. HEK293T cells were co-transfected with full-length or truncated promoter firefly-luciferase reporter constructs and the pRL-TK plasmid. Cells were lysed 24 h post-transfection. To evaluate the effects of different transcription factors on promoter or enhancer activity, the designated promoter or enhancer reporter plasmids and the pRL-TK plasmid were co-transfected into HEK293T cells along with either the empty pCAGGS vector or expression plasmids encoding specific transcription factors. Cells were lysed 24 h post-transfection. Firefly luciferase and Renilla luciferase activities were assessed using the Dual-Luciferase Reporter Assay System (Promega) following the manufacturer’s instructions. The data were presented as relative firefly luciferase activities normalized to Renilla luciferase activities and represent the average of three independent experiments.

### Plaque assays

The infectious titers of influenza viruses were assessed using plaque assays, following previously established protocols (29).

### High-throughput sequencing

A549 cells were infected with A/WSN/1933 (H1N1) at an MOI of 3 or left uninfected, followed by whole-transcriptome high-throughput sequencing conducted by Gene Denovo Biotechnology in Guangzhou, China. Total RNA was extracted from the samples, with ribosomal RNA removal to enhance the enrichment of coding RNA and non-coding RNA components. The isolated RNA was fragmented randomly into short segments, and the first cDNA strand was synthesized using six-base random primers (random hexamers). Subsequently, a mixture containing buffer solution, dNTPs (consisting of dUTP instead of dTTP), RNase H, and DNA polymerase I was employed for the generation of the second cDNA strand. The resultant cDNA was purified using the QiaQuick PCR kit and eluted with EB buffer. Following end repair, addition of base A, and incorporation of sequencing adapters, the second strand underwent digestion using Uracil-N-Glycosylase (UNG) enzyme. Fragment size selection was conducted via agarose gel electrophoresis, followed by PCR amplification. The constructed sequencing library was sequenced on the Illumina HiSeq 4000 platform.

In small RNA sequencing, total RNA is extracted from the sample utilizing the TRIzol method or CTAB method. Polyacrylamide gel electrophoresis (PAGE) is then employed to excise gel bands in the 18–30 nt range to isolate small RNA fragments. The 3′ and 5′ adapters are ligated to the small RNA molecules, followed by reverse transcription and PCR amplification. Subsequently, small RNAs linked to both adapters are purified by extracting a 140 bp band using PAGE gel, which is then eluted in EB solution to finalize library construction. Prior to sequencing, the constructed library undergoes quality and yield assessment through Agilent 2100 and ABI StepOnePlus Real-Time PCR System (Life Technologies). The high-throughput sequencing data can be accessed in Table S1.

### Microarray data sets

Microarray data sets containing IAV infection data from reputable sample sources were retrieved from the Gene Expression Omnibus (GEO) database (https://www.ncbi.nlm.nih.gov/geo/) using the GEOquery package within the R software (version 4.0.0) (30). The raw data obtained were transformed into an expression matrix, and further processing involved background correction and normalization using the limma package (31). Comprehensive details regarding the selected microarray data sets can be found in Table S1 within the supplementary materials.

### Identification of differentially expressed genes (DEGs)

Initially, DEGs were detected utilizing the limma package. Venn analysis and volcano plots of the DEGs were generated using the ggplot2 package. DEGs were identified based on adjusted *p* value < 0.05 and |log2FC| > 1 as cutoff criteria. The RRA method was employed for the integrated analysis of the gene expression profiles. The RobustRankAggreg package, necessary for this analysis, was sourced from the Comprehensive R Archive Network at https://cran.r-project.org/ (32).

### Weighted gene co-expression network analysis (WGCNA) and module preservation

The R package “WGCNA” was utilized to construct a weighted correlation network aimed at identifying key modules associated with IAV infection (33). The Pearson coefficient was calculated to assess the weighted co-expression relationships among all genes in the network. A soft threshold was applied to establish a scale-free network, and the interconnectedness of the network was evaluated using the topological overlap measure. Gene modules, comprising highly correlated genes, were identified through hierarchical clustering. Subsequently, key modules exhibiting significant correlations with IAV infection were pinpointed. The protein-protein interaction network of these key modules was constructed using the Search Tool for the Retrieval of Interacting Genes database (https://string-db.org/), with a confidence score threshold set at >0.7 for significance. In the Cytoscape software, the cytoHubba plug-in and CluoGO + Cluepedia were utilized to identify hub clusters within the significant modules and engage in functional annotation, respectively (34, 35).

### Animal experiments

The miR-302b-3p agomir and NC mimics were synthesized by GenePharma and dissolved in sterile phosphate-buffered saline (PBS). Female BALB/c mice (6–8 weeks old, sourced from Shanghai BK/KY Biotechnology Co.) were randomly allocated into four groups: NC mimics + PBS (*n* = 11), miR-302b-3p agomir + PBS (*n* = 11), NC mimics + IAV (*n* = 14), and miR-302b-3p agomir + IAV (*n* = 14). The mice received injections of agomir or mimics (100 µL) at a concentration of 100 nM/kg body weight via tail vein for three consecutive days. Infection experiments were conducted 4 days after the third tail vein injection. Three mice injected with agomir or mimics were sacrificed, and their lung tissues were processed for protein detection. Subsequently, 11 mice in each group were intranasally infected with 50 µL 0.7 × 10^4^ PFU A/WSN/1933 (H1N1), and their body weight was monitored for 14 days. Additionally, mice exhibiting greater than 30% body weight loss were humanely euthanized. To assess viral growth, three mice from each group were euthanized on day 3 post-infection for lung viral load measurement using the plaque assay. Lung samples were also subjected to hematoxylin-eosin staining for pathological examination. Supernatants from the lung samples were obtained for RNA extraction.

### Statistical analysis

The statistical analysis was carried out using R software for statistical calculations (version 4.2.2) or Prism8 (GraphPad). The data were subjected to analysis utilizing two-tailed Student’s *t*-test or one-way or two-way ANOVA, as determined. Significance levels were represented as follows: *, *P* < 0.05; **, *P* < 0.01; ***, *P* < 0.001; ****, *P* < 0.0001; and “ns” indicating no significance.

## RESULTS

### The miR-302 cluster exhibits species-specific regulation of influenza virus replication

**Fig 1:**
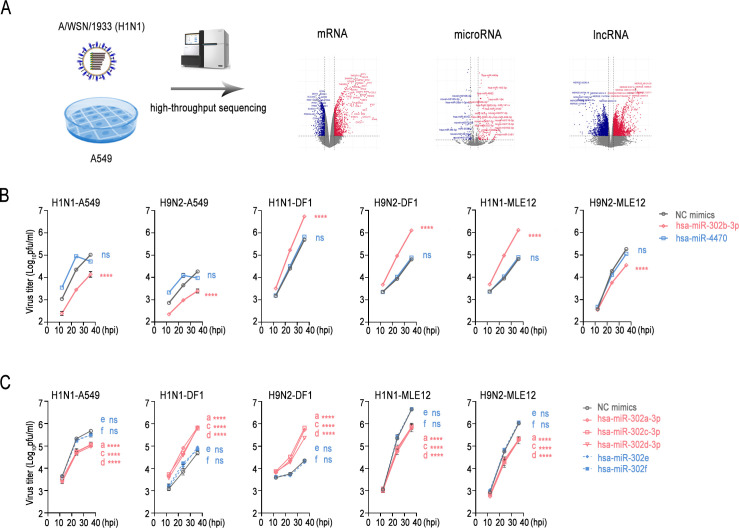
miR-302 cluster species differentially regulates influenza virus replication. (**A**) Schematic diagram illustrating the high-throughput sequencing utilized in this study. (**B**) A549, DF-1, or MLE12 cells were transfected with the indicated mimics. Cells were then infected with H1N1 or H9N2 at 24 h post-transfection (MOI = 0.01). Supernatants were collected at 12, 24, and 36 h post-infection, followed by plaque assay. (**C**) Similar to (**B**), except that other members of the miR-302 cluster were transfected into A549, DF-1, or MLE12 cells. The experiments were independently repeated three times, showing consistent outcomes. Data are presented as means ± standard deviations. Statistical differences between the overexpression group and the control group (NC mimics) are labeled according to two-way ANOVA with Dunnett’s multiple comparisons test. (a–f) indicated miR-302a-3p to miR-302f of the miR-302 cluster, respectively. ****, *P* < 0.0001; ns, no significance.

In an effort to gain a comprehensive understanding of the host-IAV interaction, this study employed whole-transcriptome sequencing to comprehensively and precisely capture coding RNAs and non-coding RNAs (including lncRNAs and microRNAs) associated with influenza virus infection (Fig. 1A; Table S1). First, we conducted a comparative analysis of the microRNA expression profiles in A549 cells infected with H1N1 (A/WSN/1933) and compared them with the reported microRNA data from A549 cells infected with H1N1 (A/California/07/2009) or H7N9 (A/Shanghai/1/2013) (Fig. S1A; Table S1) (36). Subsequent qPCR results revealed a significant downregulation of hsa-miR-135b-3p, hsa-miR-4470, hsa-miR-302b-3p, and hsa-miR-615-3p upon influenza virus infection (Fig. S1B). Transfecting A549 cells with the aforementioned microRNA mimics demonstrated remarkable inhibition of influenza virus WSN/1933 replication by hsa-miR-302b-3p (Fig. S1C). We then set out to confirm the impact of hsa-miR-302b-3p on the replication of different subtypes of influenza virus. We found that miR-302b-3p significantly stemmed the replication of influenza virus WSN/1933 (H1N1) and CK/SH/49/19 (H9N2) in A549 cells and MLE-12 cells. Instead, it promoted influenza virus replication in DF-1 cells. However, miR-4470 had no obvious effect on viral replication (Fig. 1B). Similarly, miR-302b-3p significantly suppressed the replication of influenza virus A/Hong Kong/01/1968 (H3N2) in A549 cells, while enhancing its replication in DF-1 cells (Fig. S1D). Furthermore, miR-302b-3p also inhibited the replication of influenza virus WSN/1933 (H1N1) in porcine PK-15 cells (Fig. S1E). miR-302b is a member of the miR-302 cluster (miR-302a, miR-302b, miR-302c, miR-302d, miR-302e and miR-302f). The miR-302 clusters have the same seed sequences but different non-seed flanking sequences (Fig. S1F). Surprisingly, the miR-302b-3p has exactly the same sequences among different species and is highly conserved (Fig. S1G). Next, we wondered whether the members of the miR-302 cluster had different or similar effects on influenza virus replication. The results demonstrated that, similar to miR-302b-3p, miR-302a-3p, miR-302c-3p, and miR-302d-3p significantly downregulated the viral titer of influenza virus in A549 cells and MLE12 cells. However, they promoted the replication ability of influenza virus in DF-1 cells. Notably, neither miR-302e nor miR-302f had any obvious effect on influenza virus replication in different cells (Fig. 1C). Taken together, the above results indicated that members of the miR-302 cluster differentially regulated influenza virus replication, showing a certain degree of species specificity.

### The miR-302 cluster, induced by CTNNB1, targets IRF2 to trigger IFN-induced immune responses

**Fig 2:**
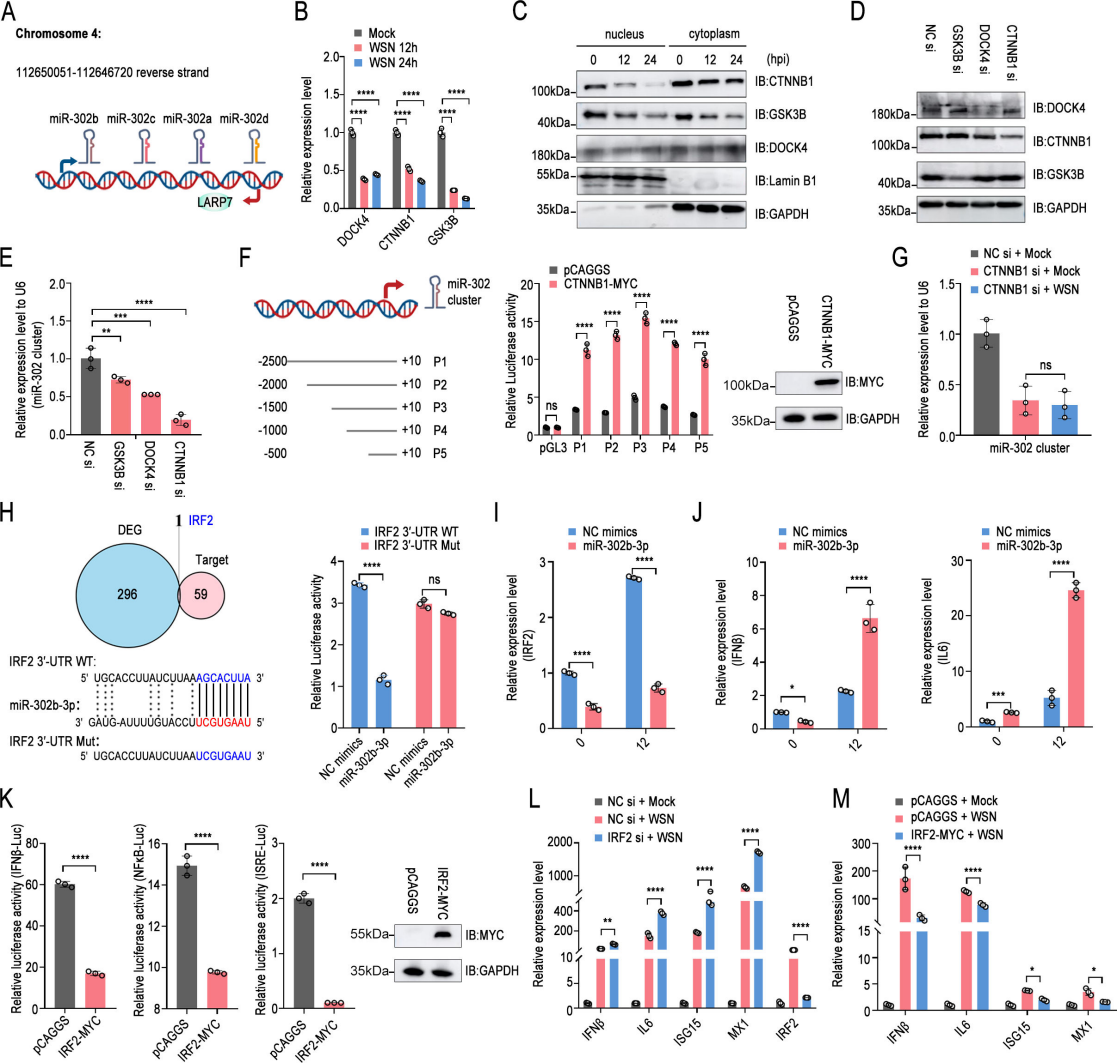
CTNNB1-induced miR-302 cluster targets IRF2, stimulating interferon-induced immune responses. (**A**) Genomic location of the miR-302 cluster on the Human genome (hg19/hg38). (**B and C**) A549 cells were either uninfected or infected with WSN/1933 (MOI = 1) and lysed at 12 and 24 h post-infection, followed by qPCR (**B**). Additionally, nuclear and cytoplasmic proteins were separately isolated for subsequent Western blot analysis (**C**). (**D and E**) A549 cells were transfected with the indicated siRNA for 48 h, followed by Western blot (**D**) or qPCR (E). (**F**) The indicated fragments were inserted into the pGL3-basic vector to generate reporter plasmids. A luciferase reporter assay was employed to measure promoter activity. The expression of MYC-tagged CTNNB1 was detected by Western blot. (**G**) A549 cells were transfected with the indicated siRNA and infected with WSN/1933 (MOI = 1) 24 h later. Subsequently, cells were lysed 24 h post-infection, followed by qPCR. (**H**) The target genes of miR-302-3p were predicted by integrating data from the miRDB, miRTarBase, and TargetScan databases with the differentially expressed genes (DEGs) from this study. A luciferase reporter assay was employed to measure miRNA target gene. (**I and J**) A549 cells were transfected with has-miR-302b-3p or NC mimics. At 24 h after transfection, cells were infected with WSN/1933 (MOI = 0.01), and then qPCR was performed to detect mRNA levels of IRF2 (**I**), IFNβ, or IL6 (**J**) at 0 and 12 h after infection. (**K**) A luciferase reporter assay was utilized to quantify the promoter activity of IFN-β, NF-κB, or ISRE element. The expression of MYC-tagged IRF2 was detected by Western blot. (**L and M**) A549 cells were transfected with siRNA targeting IRF2 or with scrambled siRNA (**L**); alternatively, cells were transfected with pCAGGS-IRF2-MYC or empty vectors for 24 h (**M**), followed by viral infection. After 12 h, the mRNA levels were evaluated via qPCR. Experiments were independently repeated three times, with similar results. The results are presented as means ± standard deviations. Statistical differences between designated groups are noted according to one-way ANOVA or two-way ANOVA with Dunnett’s multiple comparisons test. *, *P* < 0.05; **, *P* < 0.01; ****, *P* < 0.0001; ns, no significance.

The miR-302 cluster is located on the antisense strand of chromosome 4: 112650051-112646720 (GRCh38.p14). The miR-302 cluster overlaps with the La-associated protein 7 (LARP7) gene. The intergenic members miR-302e and miR-302f found in primates, located on chromosomes 11 and 18, respectively, are also classified as members of the miR-302/367 cluster (37). The miR-302 cluster is highly homologous, differing only in the 3′ hexanucleotide sequence (Fig. 2A). qPCR results showed that influenza virus infection also downregulated the transcription of miR-302a-3p, miR-302c-3p, and miR-302d-3p (Fig. S1H). It has been reported that GSK3-β/DOCK4/β-catenin in differentiated glioblastoma cells regulates the transcription of miR-302 (38). Next, we wanted to clarify the relationship between influenza virus infection and the transcription of the miR-302 cluster. The results showed that influenza virus infection downregulated the mRNA and protein levels of DOCK4, CTNNB1, and GSK3B. It is worth noting that it significantly inhibited the nuclear accumulation of CTNNB1 and GSK3B (Fig. 2B and C). Then, the above factors were further knocked down one by one (Fig. 2D), and the results indicated that the downregulation of CTNNB1 would seriously affect the transcription level of the miR-302 cluster genes (Fig. 2E). The promoter activity experiment further proved that 500 bp upstream of the miR-302 cluster transcription was sufficient to effectively initiate gene transcription, and overexpression of CTNNB1 significantly promoted the promoter activity of the reporter plasmid (Fig. 2F). In addition, knocking down CTNNB1 would offset the downregulation effect of influenza virus on the miR-302 cluster (Fig. 2G). The above results showed that the influenza virus relies on inhibiting the expression and nuclear accumulation of CTNNB1 to downregulate the transcription of the miR-302 cluster. Then, with the help of online microRNA target gene prediction tools: TargetScan, miRcode, and miRDB, the intersection only had one gene, IRF2. Dual luciferase reporter experiments further proved that miR-302b-3p can target IRF2 (Fig. 2H), and overexpression of miR-302b-3p will downregulate its mRNA level (Fig. 2I). The experiment further uncovered that the miR-302b-3p-IRF2 axis is involved in immune regulation. Transfection of miR-302b-3p stimulated the production of IFN-β and IL6 (Fig. 2J), stemming from the inhibitory effect of IRF2 on the activation of IFN-β promoter, NF-κB promoter, or ISRE elements (Fig. 2K). Knockdown of IRF2 promoted the production of ISGs (ISG15 and MX1), while overexpression inhibited them, which further proved the above point (Fig. 2L and M). Collectively, influenza virus infection downregulated CTNNB1 to interfere with the transcription of the miR-302 cluster, which targets IRF2 involved in immune response.

### The miR-302 cluster-IRFs regulate influenza virus-induced immune responses in a species-specific manner

**Fig 3:**
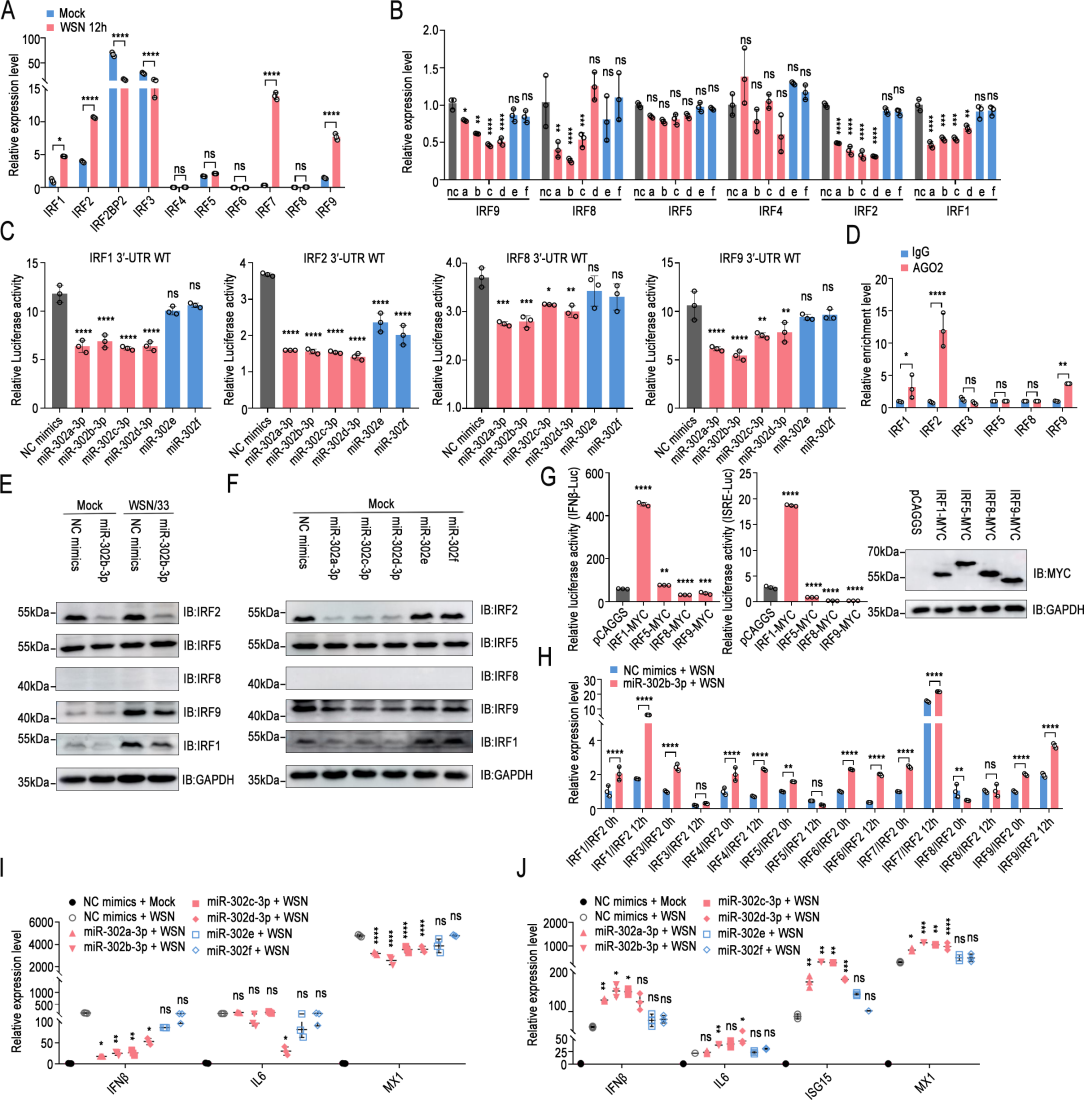
The miR-302 cluster-IRFs regulates influenza virus-induced immune responses in a species-specific manner. (**A**) A549 cells were either untreated or infected with WSN/1933 (MOI = 0.1) and lysed at 12 h post-infection, followed by qPCR. (**B**) A549 cells were transfected with miR-302 cluster members or NC mimics. After 24 h of transfection, cells were infected with WSN/1933 (MOI = 0.1) and lysed at 12 h post-infection, followed by qPCR. (a–f) indicated miR-302a-3p to miR-302f of the miR-302 cluster, respectively. (**C**) Predicted binding sites of the miR-302 cluster in the 3′-UTR of IRFs mRNA were investigated. A luciferase reporter assay was employed to measure miRNA target gene. (**D**) A549 cells were infected with WSN (MOI = 1) for 12 h. Subsequently, the infected cells were harvested and lysed for immunoprecipitation using anti-AGO2 or anti-mouse IgG antibody. The relative enrichment of each transcript was determined via qPCR. (**E**) A549 cells were transfected with miR-302b-3p or NC mimics. At 24 h post-transfection, cells were infected with WSN/1933 (MOI = 0.1) and then lysed at 12 h post-infection, followed by Western blot analysis. (**F**) A549 cells were transfected with other miR-302 cluster members or NC mimics. At 24 h post-transfection, cells were lysed and subjected to Western blot analysis. (**G**) Similar to Fig. 2K, but IRF1-MYC, IRF5-MYC, IRF8-MYC, IRF9-MYC overexpression plasmids or empty vectors were co-transfected into HEK293T cells. The expression levels of MYC-tagged IRF1, IRF5, IRF8, and IRF9 were analyzed by Western blotting. (**H**) Similar to (**E**), but the mRNA levels of each IRF at 0 and 12 h after virus infection were assessed by qPCR. In addition, IRF2 expression was utilized as a control. (**I and J**) The specified miR-302 cluster members or NC mimics were transfected into DF-1 cells (**I**) or MLE12 cells (**J**). Subsequently, 24 h post-transfection, cells were infected with WSN/1933 (MOI = 0.1) and then lysed at 12 h post-infection, followed by qPCR. The experiments were repeated independently three times, yielding consistent results. The results are presented as means ± standard deviations. Statistical differences between groups were calculated according to one-way ANOVA or two-way ANOVA with Dunnett’s multiple comparisons test, using the NC mimics or empty vectors group as controls or as specified in other panels. *, *P* < 0.05; **, *P* < 0.01; ***, *P* < 0.001; ****, *P* < 0.0001; ns, no significance.

Surprisingly, the ENCORI/starBase (Encyclopedia of RNA Interactomes) database showed that miR-302b-3p may bind to the 3′-UTR of multiple IFN regulatory factor mRNAs, including IRF1, IRF2, IRF4, IRF5, IRF8, and IRF9 (Fig. S2A). Interestingly, the mRNA binding sites of IRF1 and IRF2 in human, mouse, avian, and porcine cells are relatively conserved, while IRF9, IRF5, and RF8 are not. Importantly, the target prediction scores assigned by the computational target prediction algorithm in miRDB vary greatly, ranging from 57 to 94 (Fig. S2A), suggesting that members of the miR-302 cluster may be more complex and multifaceted in regulating cellular IFN-induced immune responses. qPCR results showed that influenza virus infection significantly induced the transcription of multiple IFN regulatory factors, including IRF1, IRF2, IRF7, and IRF9 (Fig. 3A). Then, the members of the miR-302 cluster were transfected one by one, showing that except for miR-302e and miR-302f, the other members strongly downregulated the mRNA level of IRF2 and slightly affected IRF1, IRF8, and IRF9 (Fig. 3B). Dual luciferase reporter experiments demonstrated that IRF1, IRF2, IRF8, and IRF9 were the target genes of miR-302a-3p, miR-302b-3p, miR-302c-3p, and miR-302d-3p (Fig. 3C). The results of AGO2 RIP further revealed that the miR-302 cluster-mediated RNA-induced silencing complex can bind to the mRNAs of IRF1, IRF2, and IRF9 with high affinity (Fig. 3D). Consistently, Western blot results also showed that miR-302b-3p repressed the protein levels of IRF1, IRF2, and IRF9 to varying degrees (Fig. 3E). It is worth noting that the protein level of IRF8 in A549 cells was so low that it was difficult to detect. The same phenomenon was also observed in the overexpression of miR-302a-3p, miR-302c-3p, and miR-302d-3p, but not miR-302e and miR-302f (Fig. 3F). Among the above regulatory factors, the luciferase reporter assay and qPCR results elucidated that IRF1 was the main factor that significantly activated the IFN-β promoter and ISRE element (Fig. 3G). IRF1, which positively regulates the IFN system, and conversely, IRF2, which negatively regulates, are all target genes of miR-302a-3p, miR-302b-3p, miR-302c-3p, and miR-302d-3p, instantly disrupting the relationship between the miR-302 cluster and cellular immune response. It can be speculated that the miR-302 cluster may reshape the content of intracellular IRFs, especially after viral infection. The results showed that overexpression of miR-302b-3p significantly upregulated the ratios of IRF1/IRF2, IRF3/IRF2, IRF7/IRF2, and IRF9/IRF2 (Fig. 3H), thereby rapidly activating the IFN system and inducing the transcription of downstream ISGs. As expected, miR-302a-3p, miR-302b-3p, miR-302c-3p, and miR-302d-3p activated the transcription of ISG15 and MX1 in A549, but miR-302e and miR-302f did not (Fig. S2B). Different from A549 cells, miR-302a-3p, miR-302b-3p, miR-302c-3p, and miR-302d-3p significantly downregulated the mRNA and protein levels of IRF1 in avian DF-1 cells, and slightly inhibited the protein level of IRF2, but miR-302e and miR-302f did not (Fig. S2C and E). qPCR results further showed that miR-302a-3p, miR-302b-3p, miR-302c-3p, and miR-302d-3p inhibited the production of IFN-β and ISGs in DF-1 (Fig. 3I), which also explains why they promote the replication of influenza virus in avian cells. Interestingly, in mouse MLE12 cells, members of the miR-302 cluster showed similar effects and functions as those in A549 cells (Fig. S2D and F), where miR-302a-3p, miR-302b-3p, miR-302c-3p, and miR-302d-3p significantly stimulated the transcription of ISGs (Fig. 3J). In addition, in porcine PK-15 cells, overexpression of miR-302b-3p markedly reduced IRF2 protein levels and moderately suppressed IRF1 and IRF9 protein expression (Fig. S2G), subsequently leading to a significant increase in virus-induced IFN production (Fig. S2H). The above results indicated that the miR-302 cluster-IRFs axis precisely and complexly regulated the transcription of cellular IFN-β and the production of ISGs.

### The miR-302 cluster-IRFs axis activates the transcription of hub genes and hub lncRNAs

**Fig 4:**
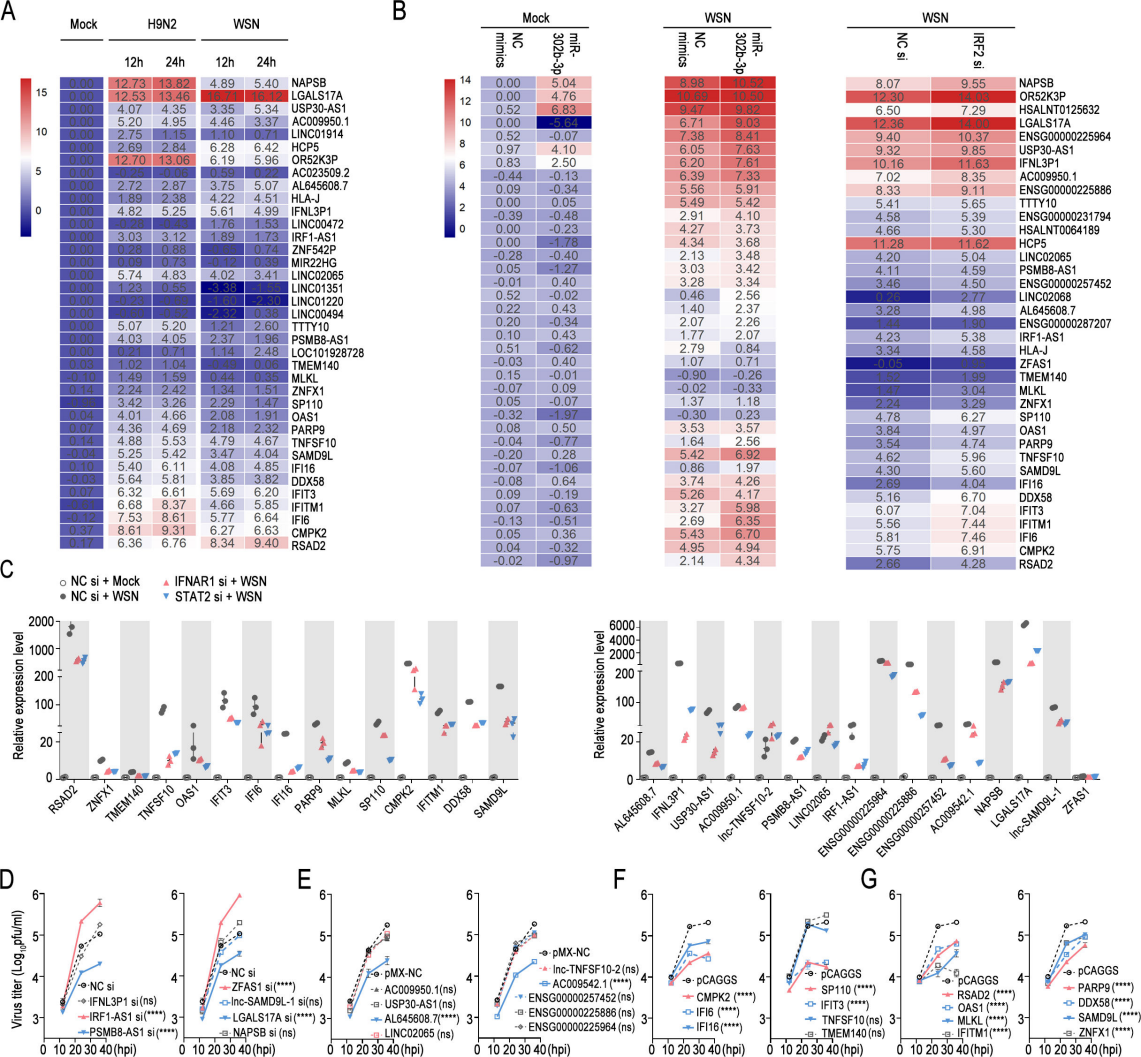
The miR-302 cluster-IRFs axis activates the transcription of hub genes and hub lncRNAs. (**A**) A549 cells were either uninfected or infected with A/WSN/1933 or CK/SH/49/19 at MOI = 0.1, and harvested at 12 and 24 h post-infection, followed by qPCR. A heat map was employed, with each row representing one gene, and the number in each rectangle indicating the log2FC value. (**B**) A549 cells were transfected with NC mimics or has-miR-302b-3p, or with the IRF2 siRNA. At 24 h post-transfection, cells were either uninfected or infected with WSN/1933 (MOI = 0.1), and harvested at 12 h post-infection, followed by qPCR. A heat map, similar to (**A**), was utilized for visualization. (**C**) A549 cells were transfected with the indicated siRNA for 24 h. Subsequently, the cells were either uninfected or infected with WSN/1933 (MOI = 0.1) and lysed after 12 h, followed by qPCR. (**D**) A549 cells were transfected with siRNA targeting specific non-coding genes or with scrambled siRNA for 24 hours, followed by infection with WSN/1933 (MOI = 0.01). Supernatants were collected at 12, 24, and 36 h post-infection, followed by plaque assay. (**E**) Similar to (**D**), but A549 cell lines overexpressing the specified non-coding RNAs constructed by the pMXs-IRES-Blasticidin retroviral vector. (**F and G**) Similar to (**D**), but A549 cells were transfected with empty pCAGGS or plasmids encoding the specified hub genes. In (D–G), statistical differences among groups were shown according to two-way ANOVA with Dunnett’s multiple comparisons test, using NC si, pMX-NC, or pCAGGS groups as controls. In addition, the statistical analysis results of (D–G) are indicated in brackets. ****, *P* < 0.0001; ns, no significance.

Next, we sought to identify novel coding and non-coding genes downstream of the miR-302 cluster-IRFs axis that play a role in regulating influenza virus replication. First, WGCNA was conducted to analyze the coding gene expression profiles of A549 cells (GSE31471, refer to Table S1 for details of microarray data sets). Hierarchical clustering analysis was conducted based on the weighted correlation, and the resulting clusters were segmented according to specified criteria to identify distinct gene modules, each represented by branches and assigned different colors on the clustering tree (Fig. S3A through D). Functional enrichment analysis of the genes within the blue modules revealed that influenza virus-infected cells predominantly activated genes associated with the NF-kappa B signaling pathway and JAK-STAT signaling pathway (Fig. S3E). Next, we used the plug-in cytoHubba in Cytoscape software to search for the hub gene that drives the above cellular responses. A total of 15 candidate hub genes were obtained (Fig. S3F). Next, for non-coding genes, we utilized the RRA algorithm to comprehensively analyze the lncRNA expression profile data (Fig. S3G, refer to Table S1 for details of the high-throughput sequencing data set). Interestingly, the blue module analyzed by “WGCNA” above also annotates many non-coding RNAs (Fig. S3H). Importantly, microarray data set GSE31471 and high-throughput sequencing data set GSE97672 further verified the high correlation between coding genes and non-coding genes obtained by the above bioinformatics analysis (Fig. S3I). qPCR results confirmed that influenza virus infection significantly upregulated all candidate coding genes and most non-coding genes (Fig. 4A). Transfection of miR-302b-3p, as a representative of the miR-302 cluster, significantly stimulated the transcription of the above coding genes and most non-coding genes. Knockdown of IRF2, the core target gene of the miR-302 cluster, showed consistent phenomena (Fig. 4B; Fig. S4C). Based on the above qPCR results, the expression correlation of candidate coding genes and non-coding genes was further analyzed, and thus, 15 key coding genes and 16 hub long non-coding RNAs were defined, which were activated by the miR-302 cluster-IRFs axis (Fig. S4A and refer to Table S1 for annotation information on the hub genes). Interestingly, most of the above hub long non-coding RNAs were located in the cell nucleus, and their relative expression levels in uninfected cells were very low (Fig. S4B). The miR-302 cluster-IRFs axis activates IFN-β and IFN-stimulated immune responses. In order to investigate the link between Hub genes and Hub lncRNAs and the IFN-β-JAK-STAT pathway, we employed siRNAs to silence IFNAR1 or STAT2 (Fig. S4C). The outcomes indicated that interference with IFNAR1 and STAT2 significantly reduced the transcription levels of hub genes and hub lncRNAs to different extents (Fig. 4C), indicating that the miR-302 cluster-IRFs axis regulated hub genes and hub lncRNAs through the IFN-β-JAK-STAT pathway.

Several hub genes have been identified to play a role in regulating the replication of RNA viruses, such as the influenza virus (39, 40). However, the functions of most hub lncRNAs are still unclear. For a detailed examination of the functions of each hub lncRNA, individual hub lncRNAs were silenced one by one using siRNAs (Fig. S4D). In cases where siRNAs were ineffective, corresponding overexpression cell lines were created utilizing lentiviral vectors (Fig. S4E). To elucidate the roles of hub genes, expression plasmids containing each hub encoding gene with MYC tags were generated (Fig. S4F). Silencing of IRF1AS1 or ZFAS1 promoted influenza virus replication, while knockdown of PSMB8-AS1 or LGALS17A demonstrated inhibitory effects during the late infection stage (Fig. 4D). Regarding the overexpressing cell lines of hub lncRNAs, AL645608.7 and AC009542.1 notably decreased the virus titer (Fig. 4E). Surprisingly, only TNFSF10 and TMEM140 among the hub genes showed no significant impact on viral replication, whereas the remaining genes markedly decreased the virus titer (Fig. 4F and G). Taken together, the miR-302 cluster-IRFs axis stimulates the transcription of downstream hub genes and hub lncRNAs, most of which inhibit influenza virus replication.

### IRF1AS forms an enhancer cluster, coordinates and *cis*-regulates the transcription of IRF1, and activates the IFN system

**Fig 5:**
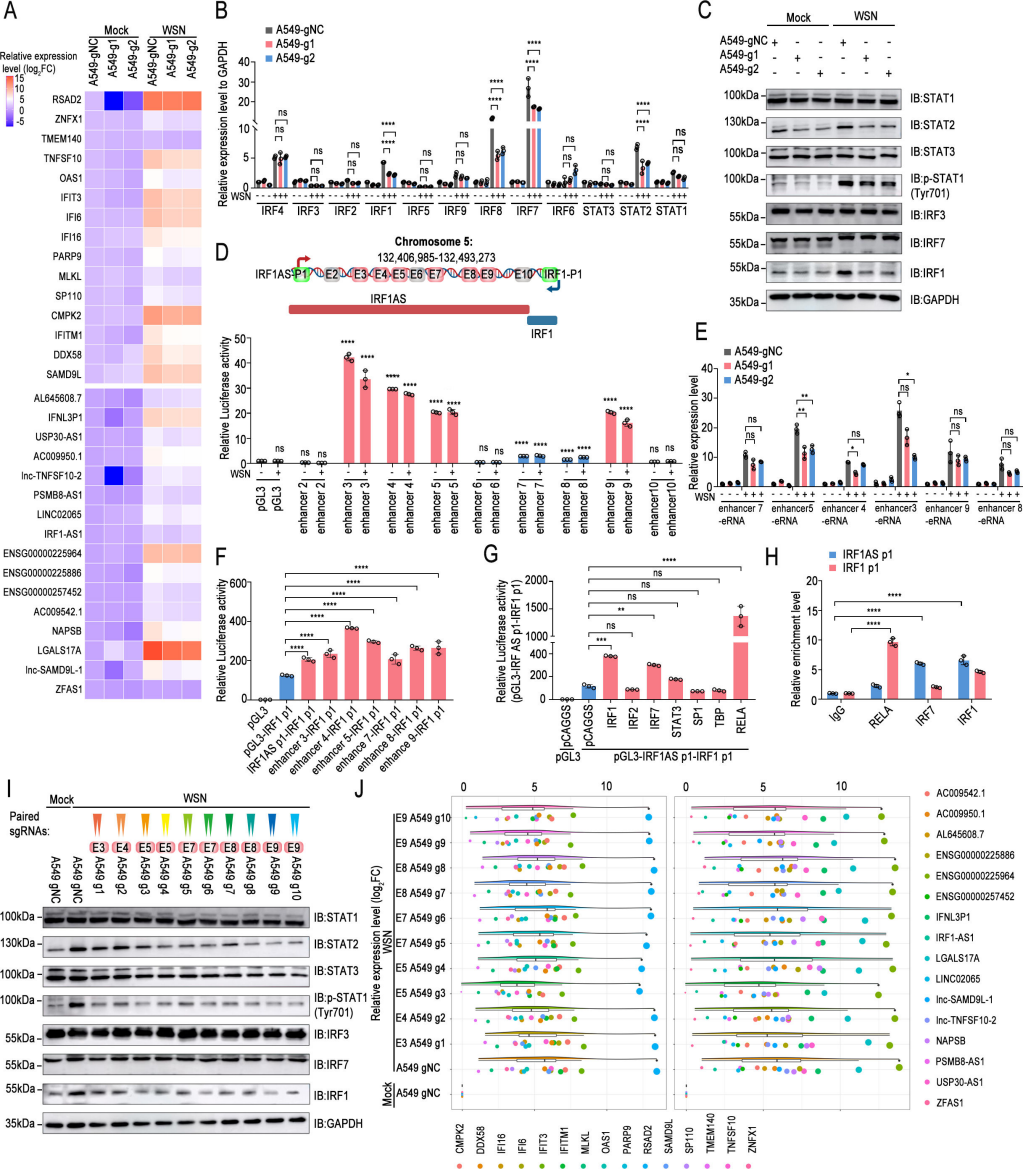
IRF1 AS forms an enhancer cluster, coordinates and *cis*-regulates the transcription of IRF1, and activates the interferon system. (A–C) A549-gNC, A549-g1, and A549-g2 cell lines were untreated (Mock) or infected with WSN/1933 (MOI = 0.1), and lysed after 12 h, followed by qPCR (**A and B**) or Western blot analysis (**C**), with color depth representing the log2FC (**A**). (**D**) Specific genomic fragments were individually amplified and cloned into the pGL3 vector (for detailed fragment information, see Table S2, eRNA analysis). A luciferase reporter assay was performed to assess enhancer activity in both uninfected and influenza virus WSN/1933-infected cells (MOI = 0.1). In the upper panel, E2–E10 correspond to enhancers 2 through 10, respectively. IRF1AS-P1 and IRF1-P1 denote the P1 fragments of the IRF1AS and IRF1 promoters, respectively, as shown in Fig. S6. (**E**) Following the procedure described in (**A**), the RNA levels of specific enhancer RNAs were assessed by qPCR. (**F**) The designated enhancer fragments were individually inserted upstream of the IRF1 promoter sequence (referred to as IRF1 P1 in Fig. S6D) and cloned into the pGL3 vector to construct a series of reporter plasmids, after which promoter activity was assessed. (**G**) HEK293T cells were co-transfected with either the pGL3 vector or the pGL3-IRF1AS p1-IRF1 p1 reporter plasmid, together with the pRL-TK plasmid and either specified gene expression plasmids or an empty vector. A luciferase reporter assay was performed to evaluate the impact of various factors on promoter activity. (**H**) Chromatin was isolated from A549 cells and subjected to immunoprecipitation with anti-RELA, anti-IRF7, anti-IRF1, or IgG antibodies, followed by ChIP. The enrichment of IRF1AS and IRF1 promoter regions was assessed using qPCR. (**I and J**) The upper panel illustrates that paired sgRNAs were designed to target different enhancer regions of IRF1AS, and knockout cell lines (A549g1 to A549g10) were subsequently generated using the CRISPR-Cas9 system. A549-gNC and the knockout cell lines were either left uninfected or infected with WSN/1933 (MOI = 0.1), lysed after 12 h, and subjected to Western blot analysis (**I**) or qPCR (**J**). In (**J**), the left panel displays the relative expression levels of hub genes, while the right panel shows the expression levels of hub lncRNAs. The mean change (log_2_FC) in each gene’s expression is depicted using a cloud plot, which combines features of a half-violin plot and a scatter plot and was generated using ggplot2. The results are presented as means ± standard deviations. In panels (D–G), statistical differences among groups were shown according to one-way ANOVA or two-way ANOVA with Dunnett’s multiple comparisons test, using Mock, pGL3-IRF1 p1, and empty vectors as control groups, respectively, and other figures were compared according to the indicated groups. *, *P* < 0.05; **, *P* < 0.01; ***, *P* < 0.001; ****, *P* < 0.0001; ns, no significance.

Within the hub genes and hub lncRNAs, multiple IFN regulatory factors, especially IRF1 and IRF2, serve as a direct target gene of the miR-302 cluster, with IRF1AS1, the non-coding RNA on its antisense strand, as a downstream target gene. This prompted the inquiry into whether they fulfill a central role in the miR-302 cluster-IRFs regulatory network. The results showed that after influenza virus infection, the transcription levels of hub genes and hub lncRNAs in IRF1AS1 exon 1 and exon 2 knockout A549 cell lines decreased sharply to varying degrees compared with the control (Fig. 5A; Fig. S5A and B). Concurrently, the transcription levels of several IFN regulatory factors, specifically IRF1, IRF7, and IRF8, were observed to decrease (Fig. 5B), alongside disruptions in the phosphorylation level of STAT1 (Fig. 5C). Interestingly, according to data from the Encyclopedia of DNA Elements (ENCODE) database (41, 42), multiple regions between the IRF1AS1 and IRF1 gene locus exhibited enhancer-like histone modifications, including H3K4me1 and H3K27ac. Upon mining the publicly available database eRNAbase (43), the examination yielded a surprising discovery: a substantial quantity of recently generated short eRNAs were transcribed in the genomic region: chr5: 132,406,985–132,493,273 (refer to the eRNA search results in Table S2 for details), predominantly comprising sequences less than 500 nucleotides in length (Fig. S5C). Additionally, noteworthy variations were observed in the transcriptional counts among different genomic regions (Fig. S5D). Significantly, eRNAs were abundantly transcribed in IRF1AS intron 1, IRF1AS intron 2, IRF1AS intron 3, IRF1AS intron 4, and the Overlap regions (Fig. S5E), corresponding to chromosome 5: 132,411,041–132,419,821, 132,419,938–132,449,646, 132,449,685–132,475,661, 132,476,038–132,481,601, and 132,481,601–132,488,694 (Fig. S5F). Delta.EPI, based on 3D genome data (44), indicated that the specified regions exhibited elevated levels of histone modifications H3K4Me1 and H3K27Ac and displayed a tendency to be DNase-sensitive, indicating their status as active enhancer regions (Fig. S5G).

Subsequently, we investigated whether the non-coding RNA IRF1AS gene locus functions as an enhancer and examined the presence of enhancers in multiple regions. Initially, promoter activity reporter experiments revealed that a region spanning 10 bp downstream to 500 bp upstream of the transcription start site of IRF1AS was capable of initiating transcription effectively, mirroring the behavior observed with IRF1 (Fig. S6A and D). Transcription factor scanning experiments revealed that IRF1 and IRF7 markedly increased the transcriptional activity of the IRF1AS promoter (Fig. S6B and C), with RELA significantly enhancing the activity of the IRF1 promoter (Fig. S6E and F). Based on the peak regions of eRNAs transcription, the genomic region chr5: 132,406,985–132,493,273 was subsequently subdivided into nine segments, designated E2–E10, along with the promoters of the IRF1AS and IRF1 genes (for details, refer to the eRNA analysis results in Table S2). Dual luciferase reporter assays indicated that E3, E4, E5, and E9 exhibited significant transcriptional activity, whereas E7 and E8 demonstrated low activity. In contrast, E2 and E6 displayed no detectable transcriptional activity (Fig. 5D). Subsequent qPCR analysis confirmed that the identified regions were actively transcribing eRNAs, with viral infection notably enhancing their transcription levels (Fig. 5E). Notably, disruption of enhancer activity was observed in the region proximal to the knockout site in the IRF1-AS1 exon 1 and exon 2 knockout A549 cell lines. Subsequently, a series of reporter plasmids were constructed by fusing the aforementioned active regions upstream of the IRF1 promoter. The results demonstrated that E3, E4, E5, E7, E8, and E9 functioned as enhancers, significantly enhancing the transcriptional activity of the IRF1 promoter (Fig. 5F). Next, we found that multiple transcription factors, including STAT3, TBP, RELA, NFKB1, and SP1, significantly augmented the promoter activity of one or more enhancers (Fig. S7A through F). Importantly, IRF1, IRF7, and RELA significantly enhanced the transcriptional activity of the merged promoter of IRF1AS and IRF1 genes (Fig. 5G). They also elevated the transcriptional activity of the merged promoter involving other enhancers and the IRF1 gene (Fig. S7G through L). Subsequent ChIP-qPCR assays confirmed the direct binding of IRF1 and IRF7 to the IRF1AS promoter region, and the direct binding of RELA to the IRF1 promoter region (Fig. 5H). Next, to investigate the functional roles of each enhancer, we employed the CRISPR-Cas9 system to design a series of paired sgRNAs for the sequential knockout of enhancer regions. Sequencing analysis confirmed varying degrees of fragment deletions within the targeted enhancer regions in the knockout cell lines (for details, refer to the cell line sequencing results in Table S2). Strikingly, the knockout of each enhancer region led to a substantial disruption in IRF1 expression (Fig. 5I), indicating a potential collaborative regulatory role where these enhancers may function collectively in an enhancer cluster to regulate the transcriptional activity of the IRF1 promoter. Consistent with expectations, the phosphorylation level of downstream STAT1 was attenuated. qPCR data provided additional validation that the intergenic enhancers of IRF1AS and IRF1 play a central role in modulating the transcription of hub genes and hub lncRNAs (Fig. 5J). Taken together, these results suggested that IRF1AS acts as an enhancer cluster to facilitate the coordinated activation of IRF1 transcription and positively reinforce the immune response triggered by the miR-302 cluster.

### The miR-302 cluster regulates IAV replication through the IRFs-IRF1AS axis and, importantly, inhibits viral replication in mice

**Fig 6:**
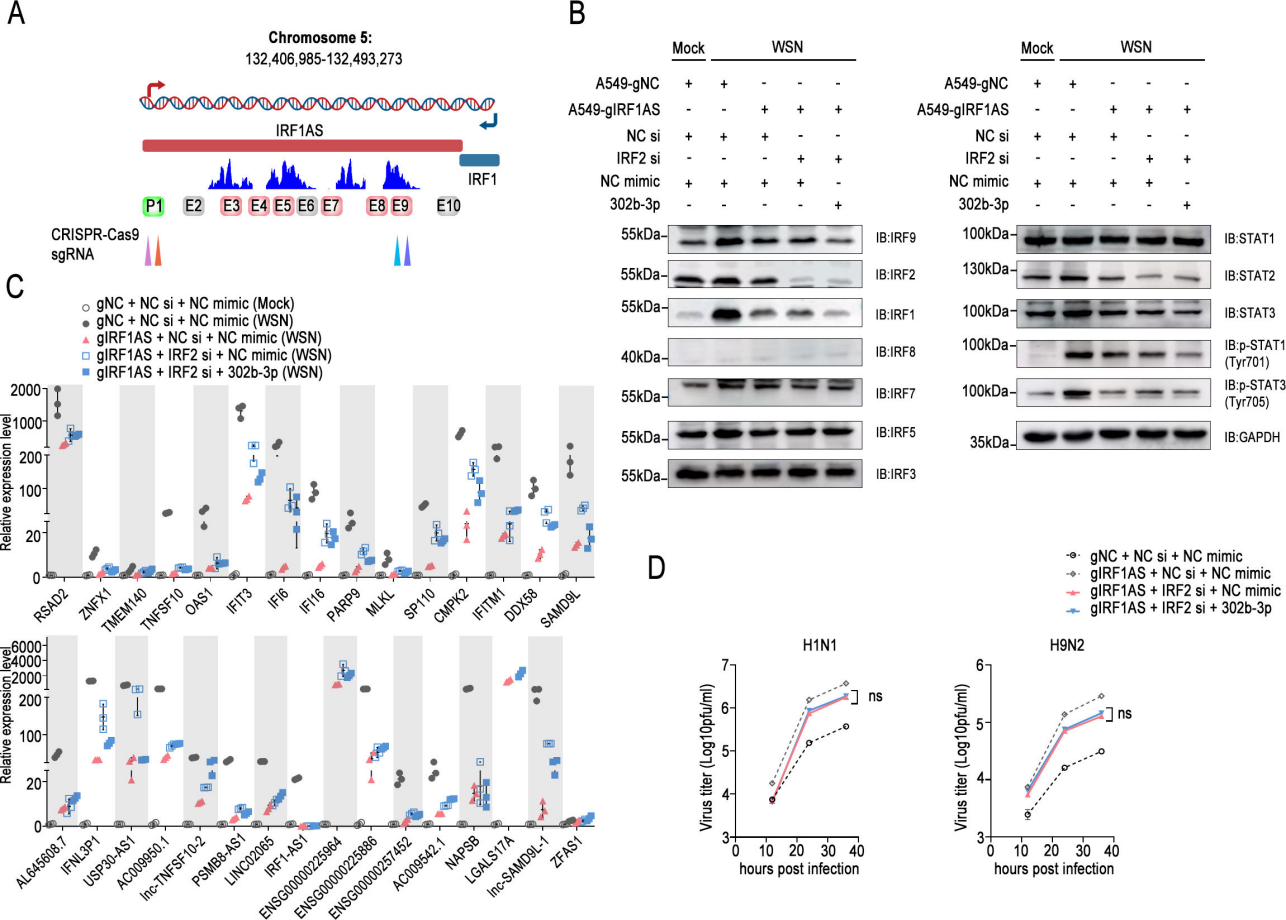
miR-302 cluster regulates replication of influenza A virus via IRFs-IRF1AS axis. (**A**) Schematic representation of the CRISPR-Cas9-mediated knockout of the IRF1AS enhancer cluster in A549 cell lines. Two pairs of sgRNAs were designed to target the IRF1AS promoter region (P1) and enhancer region (E9), thereby deleting the entire IRF1AS enhancer cluster locus. Polyclonal cell lines were selected using puromycin and validated by sequencing (see Table S3 for details). (**B and C**) A549-gNC and A549-gIRF1AS cells (IRF1AS enhancer cluster knockout cell line) were transfected with either NC siRNA or IRF2 siRNA and co-transfected with either NC mimic or miR-302b-3p. At 24 h post-transfection, the cells were infected with WSN/1933 at an MOI of 0.1, lysed after 12 h, and subjected to Western blot (**B**) or qPCR (**C**) analysis. (**D**) A549-gNC and A549-gIRF1AS cells were treated as described in (**B**) and (**C**), but were infected with either A/WSN/1933 (H1N1) or CK/SH/49/19 (H9N2) at an MOI of 0.01. Supernatants were collected at 12, 24, and 36 h post-infection and subjected to plaque assays. The experiments were independently repeated three times, yielding consistent results. Data are presented as means ± standard deviations. Statistical differences between specified groups were determined using two-way ANOVA with Dunnett’s multiple comparisons test. ns, not significant.

To further elucidate the regulatory relationship among the miR-302 cluster, IRFs, and IRF1AS, we utilized two pairs of sgRNAs to target the promoter region (P1) and the enhancer region (E9) of IRF1AS, respectively, thereby generating an A549 polyclonal cell line with a complete knockout of the IRF1AS gene locus (Fig. 6A, for sequencing details, refer to Table S2). The results demonstrated that deletion of the IRF1AS enhancer cluster in the A549-gIRF1AS knockout cell line significantly suppressed IRF1 transcription and subsequently inhibited activation of the JAK-STAT pathway (Fig. 6B). Consistently, the IRF1AS enhancer cluster knockout was confirmed to inhibit the expression of hub genes and hub lncRNAs, which we previously defined as being induced by influenza virus infection. Furthermore, additional knockdown of IRF2 in the IRF1AS-deficient cell line resulted in a slight upregulation of hub gene and hub lncRNA transcription, aligning with our previous findings. However, IRF2 knockdown reversed the miR-302b-3p-induced transcriptional activation, suggesting that the miR-302 cluster enhances the transcription of hub genes and hub lncRNAs via the IRFs-IRF1AS axis (Fig. 6C). Moreover, IRF1AS knockout significantly promoted the replication of IAV strains WSN/1933 (H1N1) and CK/SH/49/19 (H9N2), while IRF2 silencing counteracted the inhibitory effect of miR-302b-3p (Fig. 6D). These findings indicate that the miR-302 cluster regulates IAV replication through the IRFs-IRF1AS axis.

**Fig 7:**
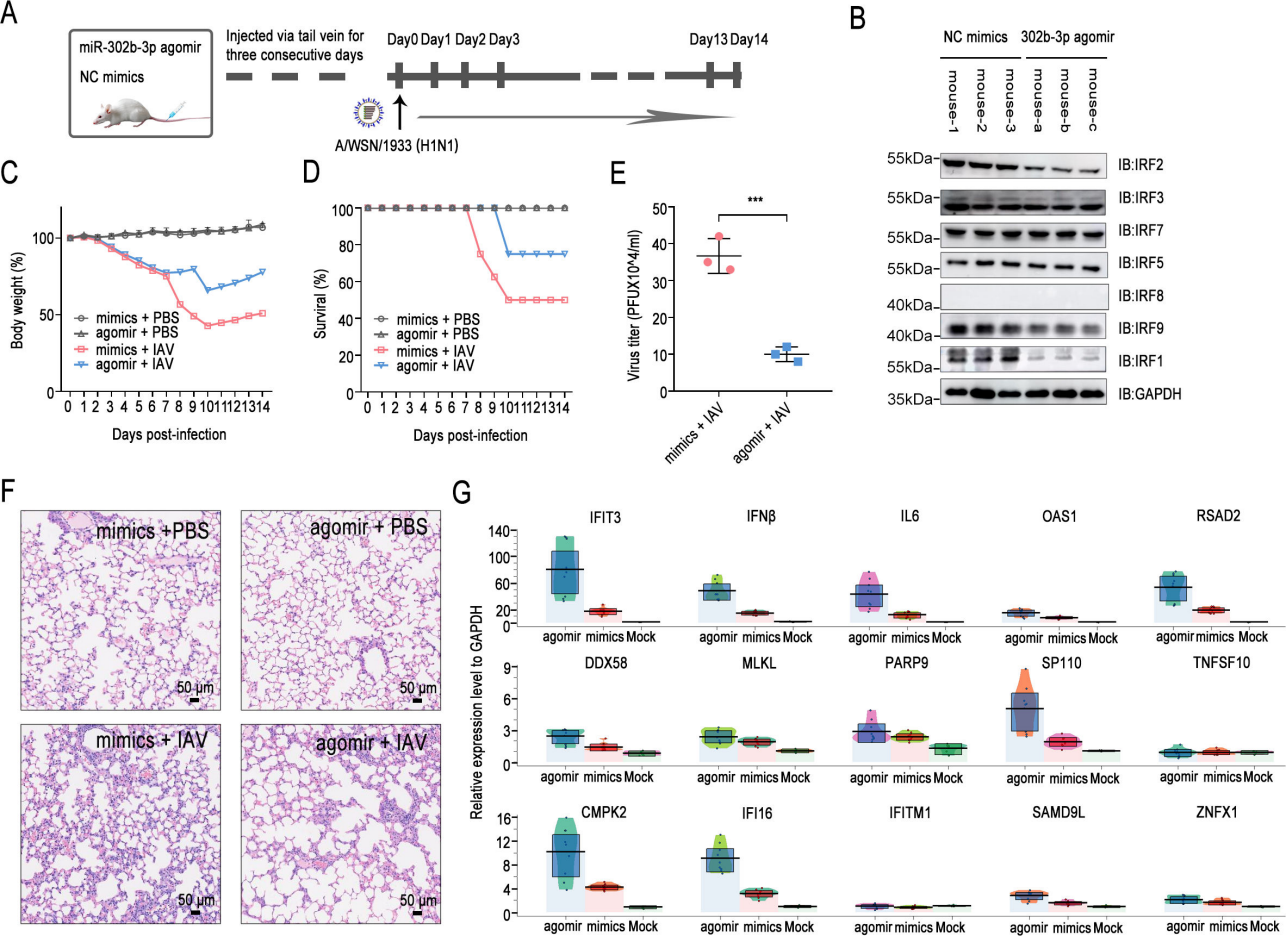
miR-302b-3p stimulates the transcription of interferon and hub genes, inhibiting influenza virus replication in mice. (**A**) Experimental design schematic for mouse studies. BALB/c mice were randomly assigned to four groups and administered miR-302b-3p agomir or NC mimics via tail vein injection for three consecutive days. (**B**) On the day of infection (Day 0), three mice from each group receiving either NC mimics or miR-302b-3p agomir were randomly selected for euthanasia. Lung tissue was collected, and the protein levels of IRFs were assessed by Western blot analysis. (**C and D**) Infection kinetics in the mice were determined by body weight loss (**C**) and the survival curve (**D**). (E–G) On the third day after infection, three mice in each group were randomly selected to be euthanized, and lung tissue was harvested. The viral loads in the lungs of mice infected with influenza virus were measured by plaque assay (**E**). Pathological sections of mouse lungs were stained with H&E. Scale bars, 50 µm (**F**). The expression levels of interferon and hub genes in lung tissue were detected by qPCR (**G**). The results are presented as means ± standard deviations. Statistical differences between designated groups are noted according to the two-tailed, unpaired *t*-test. ***, *P* < 0.001.

Subsequently, we aimed to investigate the impact of the miR-302 cluster on influenza virus replication *in vivo*. Chemically modified miRNA mimics, specifically the miR-302b-3p agomir, were synthesized for *in vivo* delivery, resulting in target gene silencing akin to the effects of endogenous miRNA overexpression. Female BALB/c mice, aged 6–8 weeks, were intravenously injected with either agomir or NC mimic for three consecutive days. Following this, the infection experiment was conducted 4 days after the third injection, with intranasal infection involving 50 µL of A/WSN/1933 (H1N1) virus (Fig. 7A). Western blot analysis demonstrated that miR-302b-3p agomir effectively reduced the protein levels of IRF1 and IRF2 in lung tissue cells of the mice and exhibited a modest effect on IRF9 (Fig. 7B). Monitoring the weight of virus-infected mice revealed that treatment with miR-302b-3p agomir partially alleviated weight loss and correlated with an improved survival rate among the mice (Fig. 7C and D). Notably, lung tissue analysis indicated that mice injected with miR-302b-3p agomir exhibited reduced inflammatory cell infiltration and lower viral load (Fig. 7E and F). Moreover, qPCR results confirmed that miR-302b-3p agomir promoted the transcription of IFN-β and downstream hub genes in response to viral infection, thereby impeding virus replication (Fig. 7G). In summary, the results suggest that the miR-302 cluster-IRFs axis plays a pivotal role in mice.

## DISCUSSION

Many miRNAs regulate host immune response signaling pathways, immune cell differentiation, and the cell cycle by targeting host immune genes. miR-485 directly targets RIG-I mRNA, resulting in its degradation (45). TRAF6 is a known target gene of miR-146a, and its overexpression in NK or T cells leads to the downregulation of TRAF6 (46). Additionally, miR-200b-3p has broad-spectrum resistance to viral infection by targeting TBK1 (47), and microRNA-200c targets contactin 1 to accelerate the degradation of MAVS and promote the replication of IAV (48). However, the roles of many other miRNAs in the innate immune response require further investigation. The miR-302 cluster plays a crucial role in cell proliferation, differentiation, and reprogramming, impacting the development of tumors, the cardiovascular system, the nervous system, and the immune system (49). Studies have reported that miR-302a inhibits the expression of IRF5, miR-302c downregulates the expression of NF-κB-inducing kinase (NIK), and miR-302b acts as an inflammatory regulator of NF-kB activation in respiratory bacterial infections (50–52). However, the association between the miR-302 cluster and influenza virus infection is still not well understood. Here, our findings indicate that the miR-302 cluster demonstrates species-specific regulation of influenza virus replication. Specifically, the overexpression of miR-302a to miR-302d resulted in a significant decrease in influenza virus titers within PK-15, A549, and MLE12 cells. Conversely, these miR-302 cluster members enhanced influenza virus replication in avian DF-1 cells, highlighting intriguing areas for further investigation.

Subsequent investigations revealed that influenza virus infection inhibited the Wnt/β-Catenin pathway. Reduced levels of CTNNB1 and GSK3B proteins, predominantly in the nucleus, substantiated this observation. Our hypothesis was further supported through promoter activity assays and dual luciferase reporter experiments, confirming the binding of nuclear protein CTNNB1 to the miR-302 cluster promoter, thereby enhancing its transcription. Surprisingly, the results of predicting the target genes of the miR-302 cluster from multiple online databases showed that members of the miR-302 cluster have the capability to target multiple interferon regulatory factors (IRF1, IRF2, IRF4, IRF5, IRF8, and IRF9). The regulatory effect of microRNA-RISC on target gene mRNA hinges on the degree of complementarity with the target gene transcript (53). Thus, we conducted an in-depth investigation into the regulatory mechanism of the miR-302 cluster-IRFs to elucidate how the miR-302 cluster demonstrates species-specific regulation of influenza virus replication. Subsequent validation through qPCR and Western blot analysis revealed significant downregulation of IRF1, IRF2, and IRF9 by miR-302a-3p, miR-302b-3p, miR-302c-3p, and miR-302d-3p, while miR-302e and miR-302f did not induce similar effects. Dual luciferase reporter assays and RIP assays further demonstrated the direct interaction of the miR-302 cluster with the mRNA of IRF2, IRF1, and IRF9. Additionally, the binding strength decreased significantly, correlating with the level of silencing observed for IRF1, IRF2, and IRF9 while also shedding light on the increased ratios of IRF1/IRF2, IRF3/IRF2, IRF7/IRF2, and IRF9/IRF2, resulting in the significant enhancement of the IFN system by the miR-302. cluster-IRFs in human A549 cells and mouse MLE12 cells, while displaying an inhibitory effect in avian DF1 cells. The discrepancy between species could be partially explained by the absence of DDX58 and IRF3 in avian cells, which renders the immune system vulnerable. The miR-302 cluster rapidly suppresses avian IRF1 mRNA while leaving IRF2 unaffected, leading to a reduction in the IRF1/IRF2 ratio. Additionally, the mRNA sequences of avian IRF5, IRF8, and IRF9 do not possess binding sites for the miR-302 cluster, thereby preventing competitive binding with it.

While there are numerous studies on IFN-β-stimulated coding genes, limited information is available about the downstream non-coding RNAs (9, 54). To delve deeper into the novel coding and non-coding genes activated by the miR-302 cluster-IRFs axis, and to address the gap in understanding the role of non-coding RNAs in the immune regulatory network, beyond analyzing the lncRNA expression profile data from this study, we conducted a comprehensive examination of transcriptomic data obtained from diverse human lung cells and tissues, such as primary human alveolar epithelial type II cells (AECIIs), alveolar macrophages (AMs), the human leukemia monocytic cell line THP-1, and the epithelial cell line A549, sourced from public databases. Additionally, we included transcriptome data from A549 cells treated with IFN-β and conducted data mining on the microarray data sets using WGCNA. Ultimately, we defined 15 crucial coding genes and 16 key long non-coding RNAs as downstream genes of the miR-302 cluster-IRFs axis. Several hub lncRNAs implicated in the regulation of influenza virus replication, such as ZFAS1, IRF1AS1, LGALS17A, AL645608.7, and AC009542.1, were identified for the first time. Furthermore, the majority of these hub genes exhibited notable inhibitory effects on influenza virus replication, some of which are already known to be associated with influenza viruses, demonstrating the reliability of our WGCNA method (55–57). However, the regulatory mechanisms of most of the above-mentioned hub lncRNAs and several hub genes (e.g., SP110, PARP9, and SAMD9L) on influenza virus replication have not been reported and need to be further elucidated.

The miR-302 cluster-IRFs axis targets IRF1, but upregulates its antisense non-coding RNA, IRF1AS1. Dual luciferase reporter and ChIP assays subsequently validated the binding of IRF1, IRF7, and RELA to the promoter region of IRF1AS1, enhancing its transcription. This suggests the existence of a possible regulatory feedback loop between IRF1 and its antisense transcript, IRF1AS1. Interestingly, analysis through the ENCODE database revealed that, apart from the IRF1AS1 promoter region, multiple regions spanning between IRF1AS1 and the IRF1 gene exhibited histone modifications characteristic of enhancers, such as H3K4me1 and H3K27ac. Furthermore, the eRNAbase database indicated a significant presence of newly transcribed short eRNAs in the genomic region chr5: 132,406,985–132,493,273, suggesting that IRF1AS may act as an enhancer. In subsequent experiments, qPCR and promoter activity assays were performed to validate the transcriptional activity of specific regions within the genomic region chr5: 132,406,985–132,493,273, including regions with high activity (E3, E4, E5, and E9) and low activity (E7 and E8), encompassing both the IRF1AS1 and IRF1 promoter regions. Subsequently, a dual luciferase reporter assay screening identified key transcription factors, such as STAT3, TBP, RELA, NFKB1, and SP1, as significant regulators of the activity of various enhancer regions. Further analysis using a fusion reporter system, combining the IRF1 promoter region with specific enhancer regions, confirmed their enhancer functionality. Importantly, deletion of any enhancer region led to a noticeable disruption in IRF1 expression, impacting IFN-β production, JAK-STAT activation, and subsequently influencing the transcription of hub genes and lncRNAs to varying degrees. These results suggest that IRF1AS acts as an enhancer cluster, orchestrating and cis-regulating IRF1 transcription to activate the IFN system. However, several questions require further investigation: (i) The genomic region chr5: 132,406,985–132,493,273, corresponding to the IRF1AS gene, contains multiple enhancer regions that form the IRF1AS enhancer cluster. Do these enhancers exhibit a hierarchical structure? (ii) What is the underlying mechanism of interaction between the IRF1AS enhancer cluster and the IRF1 promoter? (iii) What are the biological characteristics and functions of the numerous eRNAs transcribed by the IRF1AS enhancer cluster?

**Fig 8:**
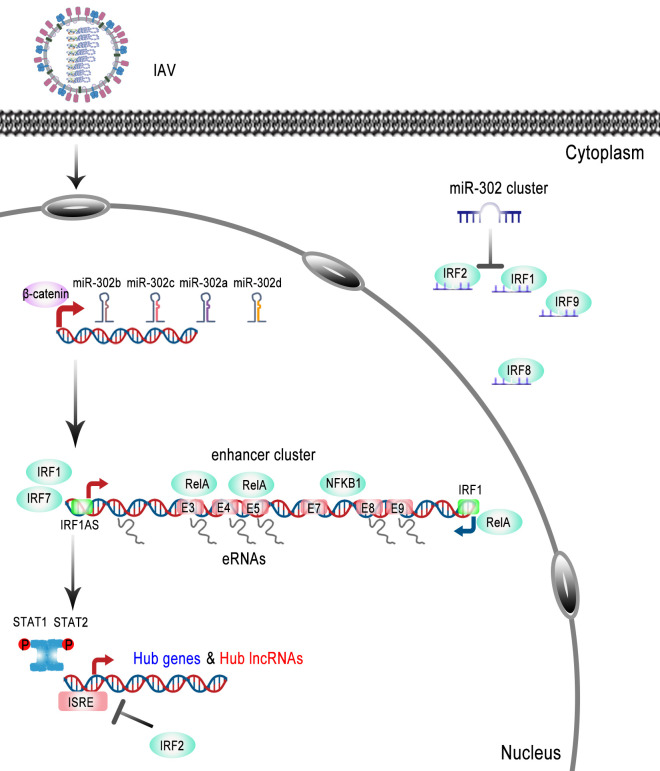
Schematic diagram of the miR-302 cluster-IRFs-IRF1AS axis regulating influenza virus replication. Influenza virus infection downregulates CTNNB1, thereby affecting the transcription of the miR-302 cluster. In turn, the miR-302 cluster targets various interferon regulatory factors (primarily IRF2, IRF1, and IRF9) with different affinities and silencing efficiencies and regulates cellular immune responses in a species-specific manner, leading to the activation of hub genes and hub lncRNAs as defined by WGCNA. Interestingly, IRF1 and IRF7 not only bind to the promoter region of IRF1AS to promote the transcription of numerous enhancer RNAs, but also, the IRF1AS locus forms an enhancer cluster that coordinates the cis-regulation of IRF1 transcription, thereby rapidly amplifying the antiviral immune response initiated by the miR-302 cluster-IRFs axis.

MicroRNA regulation is highly complex, characterized by many-to-many regulatory interactions and diverse regulatory mechanisms. Notably, the miRNA regulatory network is not static but is dynamically influenced by various factors, including cellular conditions and environmental stimuli. Our rescue experiments demonstrated that the complete knockout of the IRF1AS gene locus significantly suppressed IRF1 transcription, downregulated the expression of downstream hub genes and hub lncRNAs, and enhanced viral replication. Moreover, IRF2 silencing in IRF1AS-knockout cell lines reversed the effects of miR-302b-3p, further confirming that the miR-302 cluster regulates IAV replication through the IRFs-IRF1AS axis. In summary, we explored the relationship between the miR-302 cluster and influenza virus, highlighting the species-specific regulatory effects of the miR-302 cluster-IRFs. Furthermore, we identified several novel coding and non-coding genes involved in influenza virus replication. Notably, our findings demonstrated that IRFs and IRF1AS engage in feedback regulation during the antiviral immune response induced by influenza virus, thereby refining the regulatory network of viral infection and offering new insights into immune regulatory mechanisms as shown in Fig. 8.

## Data Availability

The authors confirm that the data supporting the findings of this study are available within the article and its supplementary materials.

## SUPPLEMENTAL MATERIAL

Supplemental figures mbio.01375-25-s0001.docx Fig. S1-S7 and captions for supplemental tables.

Table S1 mbio.01375-25-s0002.xlsx High-throughput sequencing data and microarray data sets used in this study.

Table S2 mbio.01375-25-s0003.xlsx Search results from the eRNAbase database, annotation information for eRNAs, and analysis of the IRF1AS enhancer.

Table S3 mbio.01375-25-s0004.xlsx Primers, siRNAs, ligonucleotides, antibodies, gene sequences, and cell line sequencing data.

ASM does not own the copyrights to Supplemental Material that may be linked to, or accessed through, an article. The authors have granted ASM a non-exclusive, world-wide license to publish the Supplemental Material files. Please contact the corresponding author directly for reuse.
