# Supplementary material for: The miR-302 cluster-IRFs-IRF1AS axis regulates influenza A virus replication in a species-specific manner
Source: mBio. 2025 Jul 8;16(8):e01375-25. doi: 10.1128/mbio.01375-25 (PMC12345190; doi:10.1128/mbio.01375-25)
Supplement: Supplemental figures — Fig. S1-S7 and captions for supplemental tables. [file mbio.01375-25-s0001.docx]

**Supplemental figures**

**The miR-302 cluster-IRFs-IRF1AS axis regulates influenza A virus replication in a species-specific manner**

Lingcai Zhao^1^, Chenglin Hou^1^, Xifeng Hu^1^, Chenfeng Jiang^1^, Shengmin Li^1^, Jun Xia^2^, Jihui Ping*^1^

1 MOE Joint International Research Laboratory of Animal Health and Food Safety, Engineering Laboratory of Animal Immunity of Jiangsu Province, College of Veterinary Medicine, Nanjing Agricultural University, Nanjing 210095, China;

2 Xinjiang Acadamy of Animal Sciences, Institute of Veterinary Medicine (Research Center of Animal Clinical), Urumqi 830000, China.

Lingcai Zhao, lczhao@njau.edu.cn;

Chenglin Hou, chenglinhou@163.com;

Xifeng Hu, xifhu@163.com;

Chenfeng Jiang, 296536152@qq.com;

Shengmin Li, lishengmincandy@163.com;

Jun Xia, xiajun2004263@163.com;

*Correspondence: jihui.ping@njau.edu.cn


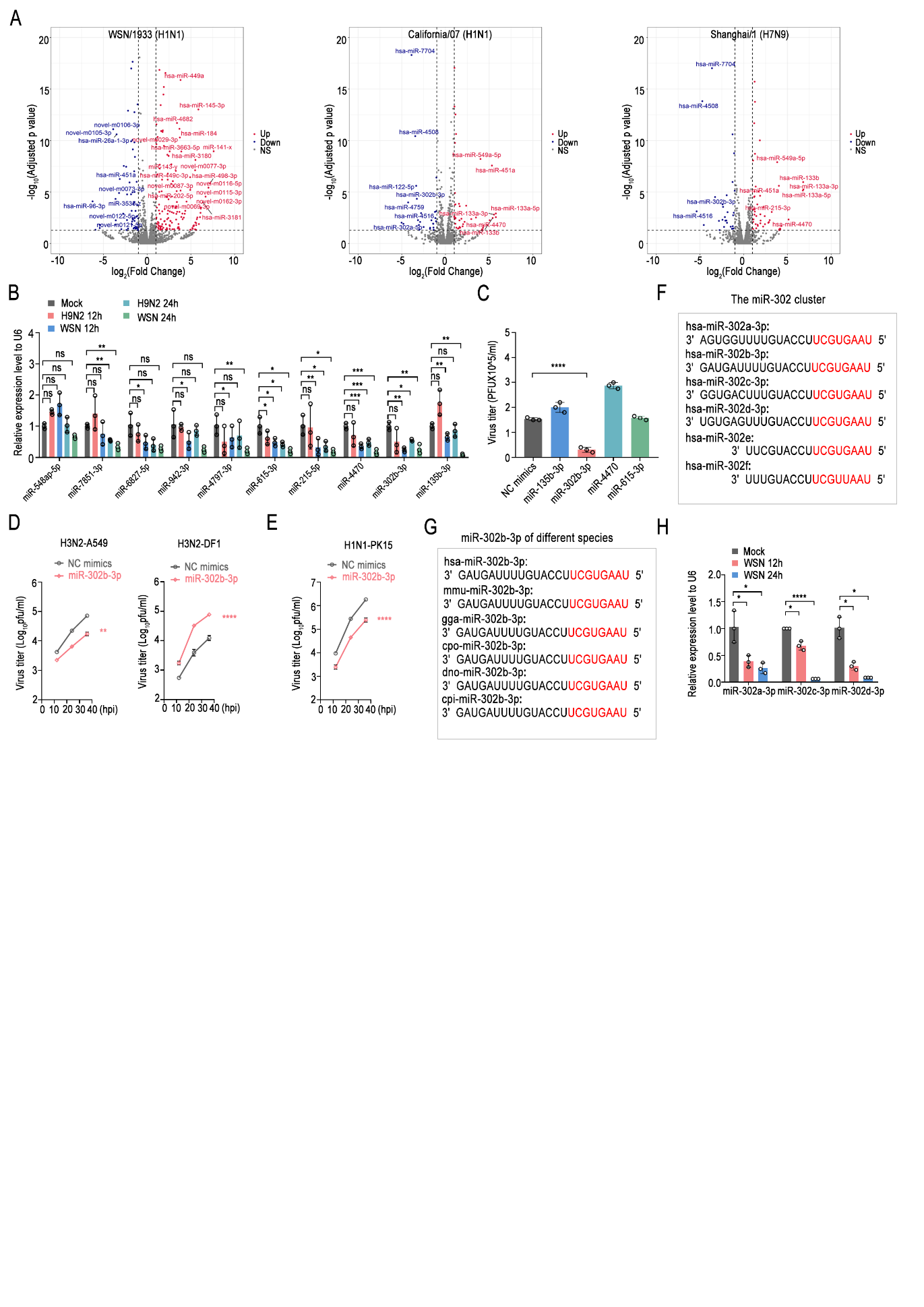


**Fig S1. Screening and verification of differentially expressed microRNAs.** (A) The volcano plot illustrates the differentially expressed microRNAs following infections with H1N1 (A/WSN/1933), H1N1 (A/California/07/2009), or H7N9 (A/Shanghai/1/2013) strains. The microRNA expression profiles in A549 cells infected with H1N1 (A/California/07/2009) and H7N9 (A/Shanghai/1/2013) were obtained from a referenced study (PMID: 35159296). In the plot, red dots signify upregulated genes (adjusted p < 0.05 and logFC > 1), blue dots denote downregulated genes (adjusted p < 0.05 and logFC < -1), and gray dots represent genes that did not exhibit significant differential expression. Statistically significant microRNAs (adjusted p < 0.05 and logFC > 1) have been highlighted. (B) A549 cells were infected with A/WSN/1933 (H1N1) or CK/SH/49/19 (H9N2) at MOI = 0.1 and collected at 12h and 24h post-infection, followed by qPCR analysis. (C) A549 cells were transfected with the indicated mimics. At 24h after transfection, cells were infected with WSN/1933 viruses (MOI = 0.01). Supernatants were collected 24 hours post-infection, followed by plaque assay. (D) A549 or DF-1 cells were transfected with the indicated mimics. Cells were then infected with H3N2 at 24 hours post-transfection (MOI = 0.01). Supernatants were collected at 12h, 24h, and 36h post-infection, followed by plaque assay. (E) Similar to (D), but PK-15 cells were infected with WSN/1933 (MOI = 0.01). (F) Sequence information of each member of the miR-302 cluster. (G) Nucleotide sequences of miR-302b-3p from Homo sapiens, Mus musculus, Gallus gallus, Cavia porcellus, Dasypus novemcinctus, and Chrysemys picta. (H) A549 cells were either uninfected or infected with WSN/1933 (MOI = 1) and subsequently lysed at 12h and 24h post-infection, followed by qPCR. The results are presented as means ± standard deviations. Statistical differences between designated groups are noted according to one-way ANOVA or two-way ANOVA with Dunnett's multiple comparisons test. *, *P* < 0.05; **, *P* < 0.01; ***, *P* < 0.001; ****, *P* < 0.0001; ns, no significance.


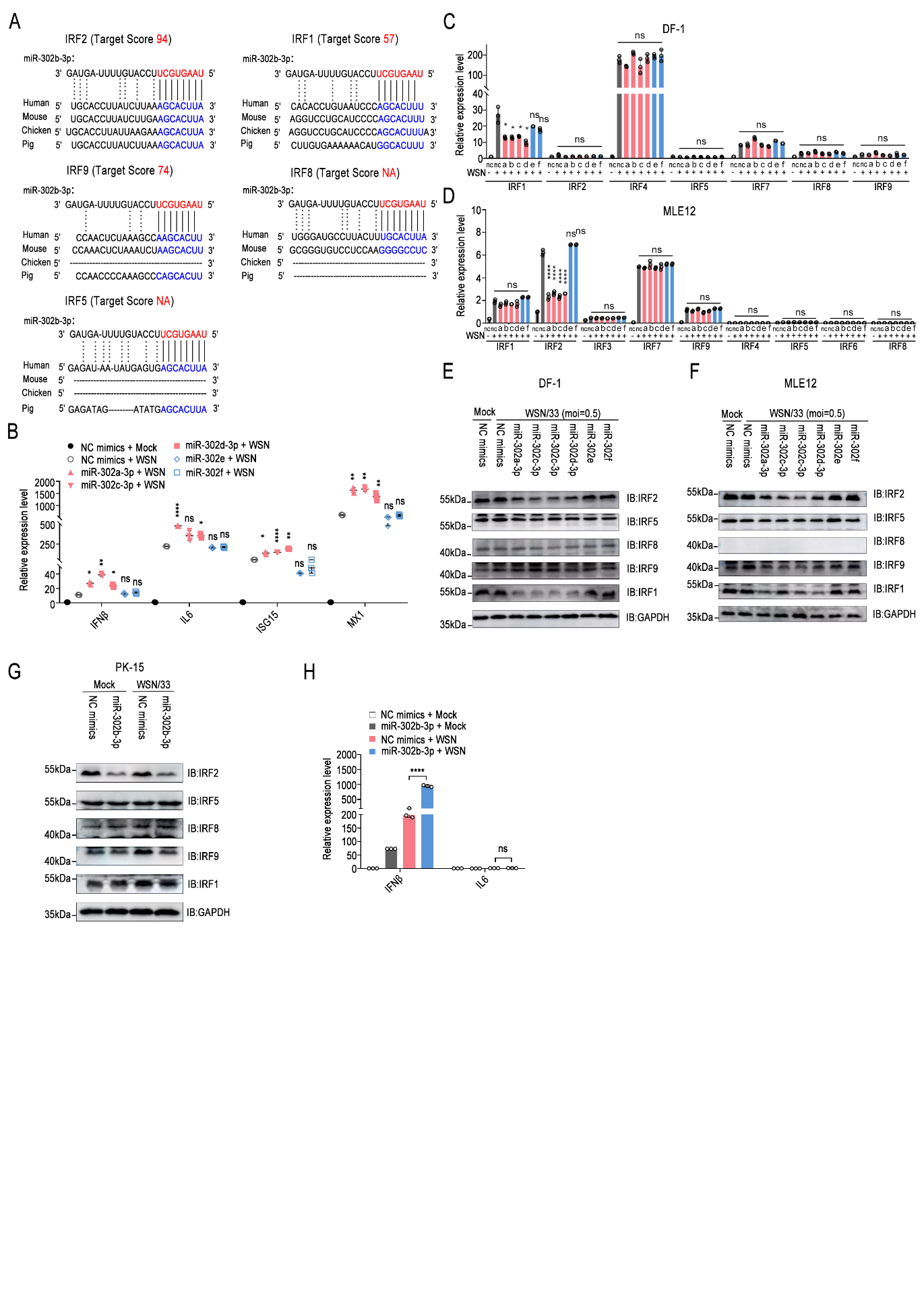


**Fig S2. Members of the miR-302 cluster exhibit species-specific targeting of multiple interferon regulatory factors.** (A) Predicted binding sites of miR-302b-3p in the 3'-UTR of various IRF mRNAs in human, mouse, chicken and pig. A solid line denotes a complete match between the seed region and the target site, while a dashed line indicates matching base pairs in the flanking region. The Target Score reflects the likelihood of hsa-miR-302b-3p binding to the target gene in the miRDB database. 'NA' indicates the absence of the target gene in the database. (B) A549 cells were transfected with the indicated mimics. At 24 hours post-transfection, the cells were infected with WSN/1933 (MOI = 0.1) and harvested at 12 hours post-infection, followed by qPCR analysis. (C, D, E and F) DF-1 cells were transfected with miR-302 cluster members or NC mimics. After 24 hours of transfection, the cells were infected with WSN/1933 (MOI = 0.1) and then harvested at 12 hours post-infection, followed by qPCR analysis (C) or Western blot analysis (E). MLE12 cells were subjected to the same treatment as described above, followed by qPCR analysis (D) or Western blot analysis (F). (G and H) PK-15 cells were transfected with miR-302b-3p or NC mimics. After 24 hours of transfection, the cells were infected with WSN/1933 (MOI = 0.1) and then harvested at 12 hours post-infection, followed by Western blot analysis (G) or qPCR analysis (H). The experiments were repeated independently three times, yielding consistent results. The results are presented as means ± standard deviations. In panels B, C, D and H, statistical differences between groups were calculated according to one-way ANOVA or two-way ANOVA with Dunnett's multiple comparisons test, using the NC mimics (WSN-infected group) as controls. a-f indicated miR-302a-3p to miR-302f of the miR-302 cluster, respectively. *, *P* < 0.05; **, *P* < 0.01; ***, *P* < 0.001; ****, *P* < 0.0001; ns, no significance.


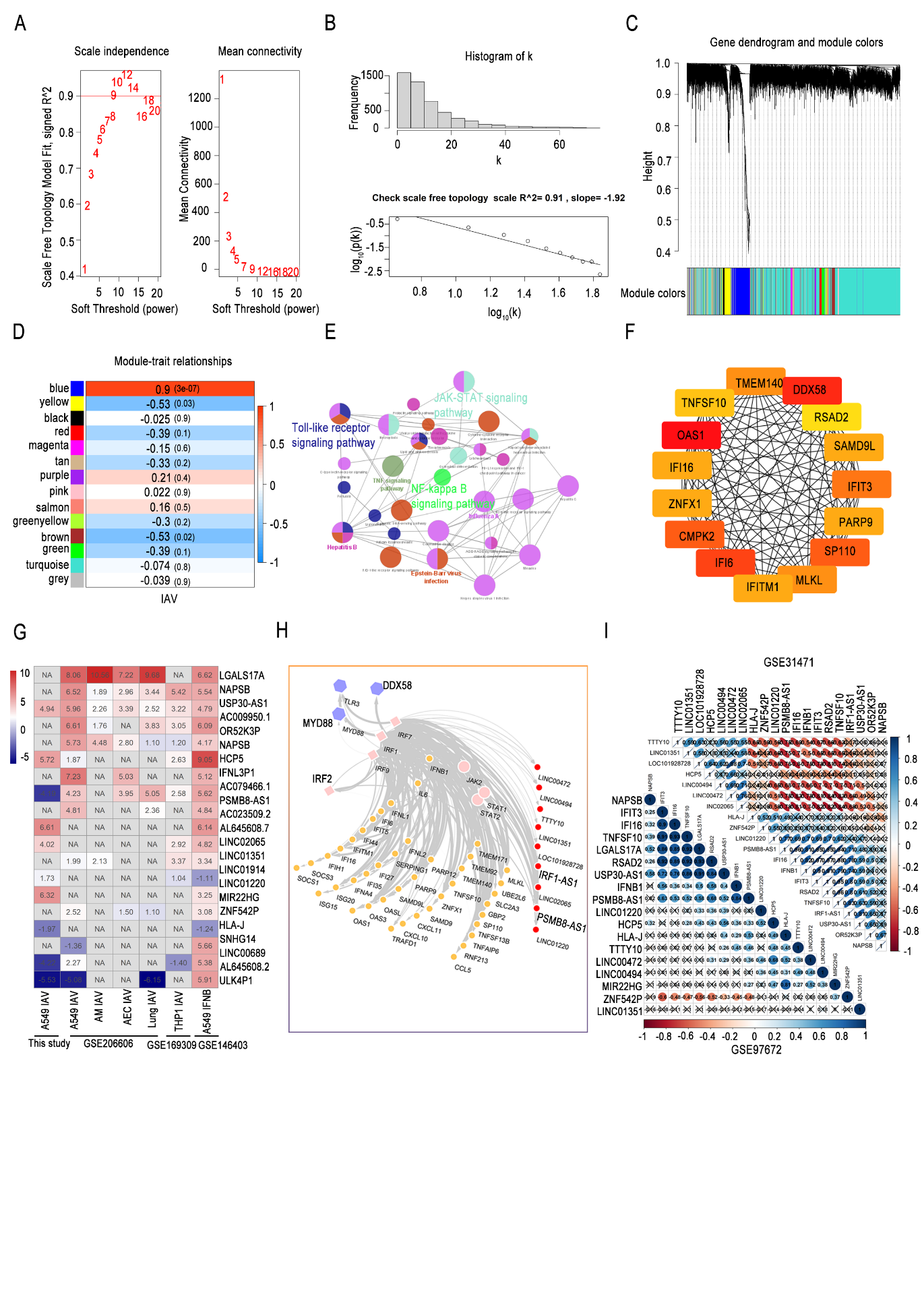


**Fig S3. Screening and identification of hub genes and hub lncRNAs.** (A) Examination of network topology at various soft thresholding powers; the scale-free fit index is shown in the left panel (y-axis) against the soft-thresholding power (x-axis), while the mean connectivity (degree) is depicted in the right panel (y-axis) in relation to the soft-thresholding power (x-axis). A power of 9 was selected for subsequent analysis. (B) Histogram and assessment of scale-free topology. (C) Cluster dendrogram illustrating co-expression network modules, with distinct colors representing different modules. (D) Analysis of module-trait relationships; each row corresponds to a colored module, and each column represents a trait (influenza virus infection-induced cellular responses). Each cell contains the correlation coefficient and the respective p-value. (E) Functional enrichment analysis was conducted on the genes within the blue and yellow modules using the ClueGO+Cluepedia plugin. (F) Hub genes within the Protein-Protein Interaction (PPI) network created by the genes in the blue and yellow modules were identified. (G) A heatmap displaying the top 19 up-regulated lncRNAs identified in the RRA analysis. High-throughput sequencing data from this study and datasets (GSE206606, GSE169309, and GSE146403) obtained from the GEO (https://www.ncbi.nlm.nih.gov/geo/) were detailed in the supplementary material. Each row corresponds to a gene, and each column represents a dataset. The numerical value in each cell indicates the log2FC. "NA" denotes data that is not available. (H) Determination of coding and non-coding genes within the Protein-Protein Interaction network established by the genes in the blue modules, as revealed through WGCNA of the microarray dataset GSE31471. (I) Analysis of expression correlations among chosen coding and non-coding genes from dataset GSE31471 or GSE97672. Blue indicates a positive correlation, while red signifies a negative correlation. The size and color intensity of the dots reflect the strength of the correlation. Each cell contains the correlation value between genes; a cross denotes non-significance (p > 0.05).


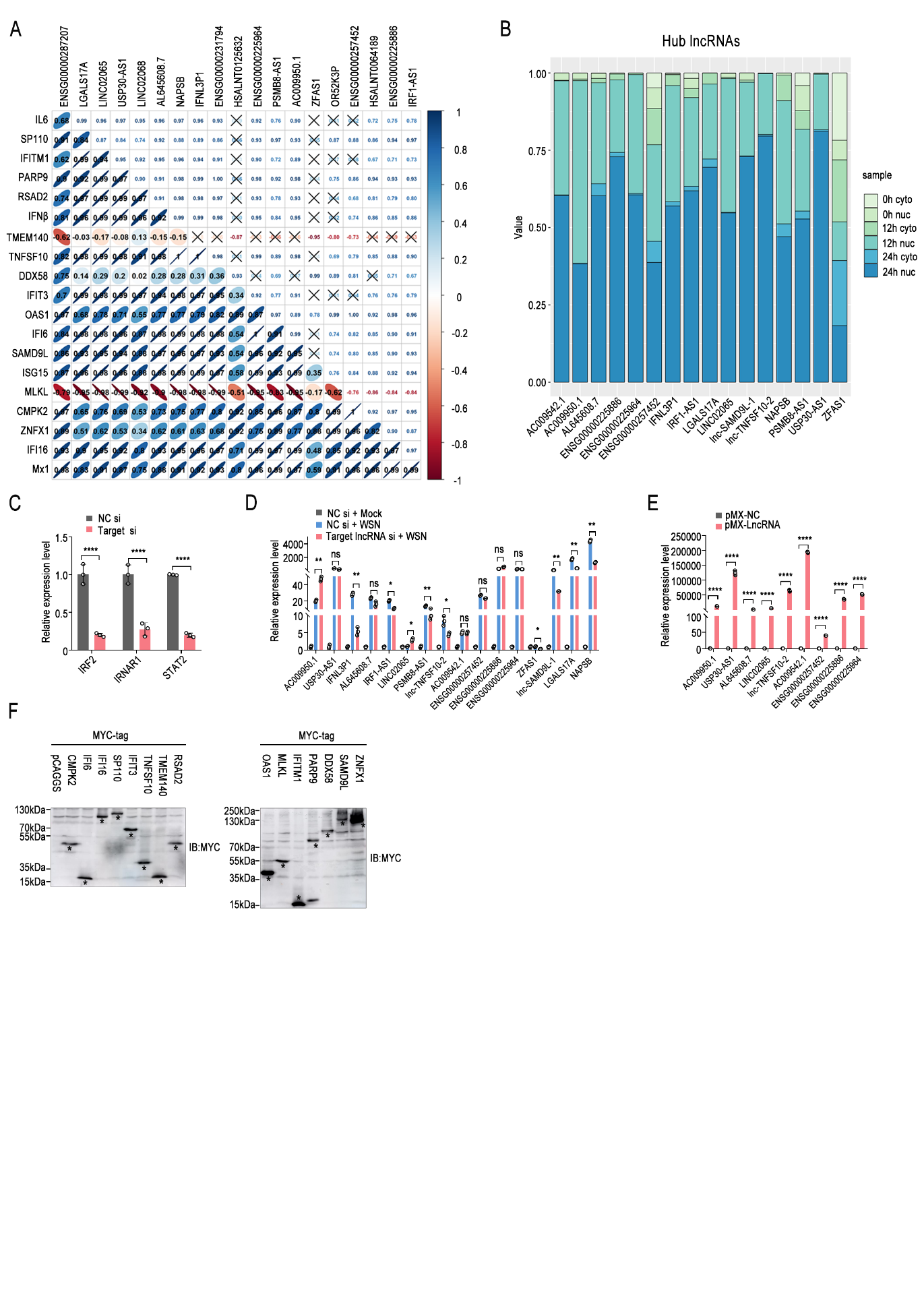


**Fig S4. The miR-302 cluster-IRFs axis activates the transcription of genes and lncRNAs.** (A) A549 cells were infected with WSN/1933 (MOI = 0.1), followed by cell lysis after 12 hours. The RNA levels of specific genes were evaluated via qPCR. Analysis of expression correlations among selected coding and non-coding genes, akin to Figure 4C. (B) A549 cells were either untreated or infected with WSN/1933 (MOI = 0.1) and lysed at 12h and 24h post-infection. Subsequently, nuclear and cytoplasmic RNA were separately isolated and subjected to qPCR analysis. (C) A549 cells were transfected with the specified siRNA, and after 24 hours, the cells were lysed. The silencing efficiency of each target gene was evaluated using qPCR. (D) A549 cells were transfected with the indicated siRNA for 24 hours. Subsequently, the cells were either left untreated or infected with WSN/1933 (MOI = 0.1), and lysed after 12 hours. The silencing efficiency of each Hub lncRNA was assessed by qPCR. (E) The pMXs-IRES-Blasticidin retroviral vector was utilized to create overexpression cell lines of specified non-coding RNAs, and the overexpression efficiency of each lncRNA was determined by qPCR. (F) A549 cells were transfected with empty pCAGGS or plasmids encoding the specified Hub genes with MYC-tag for 24 hours, followed by Western blot analysis. An asterisk (*) indicates the target protein band. Statistical differences among groups were shown according to one-way ANOVA or two-way ANOVA with Dunnett's multiple comparisons test. *, *P* < 0.05; **, *P* < 0.01; ***, *P* < 0.001; ****, *P* < 0.0001; ns, no significance.


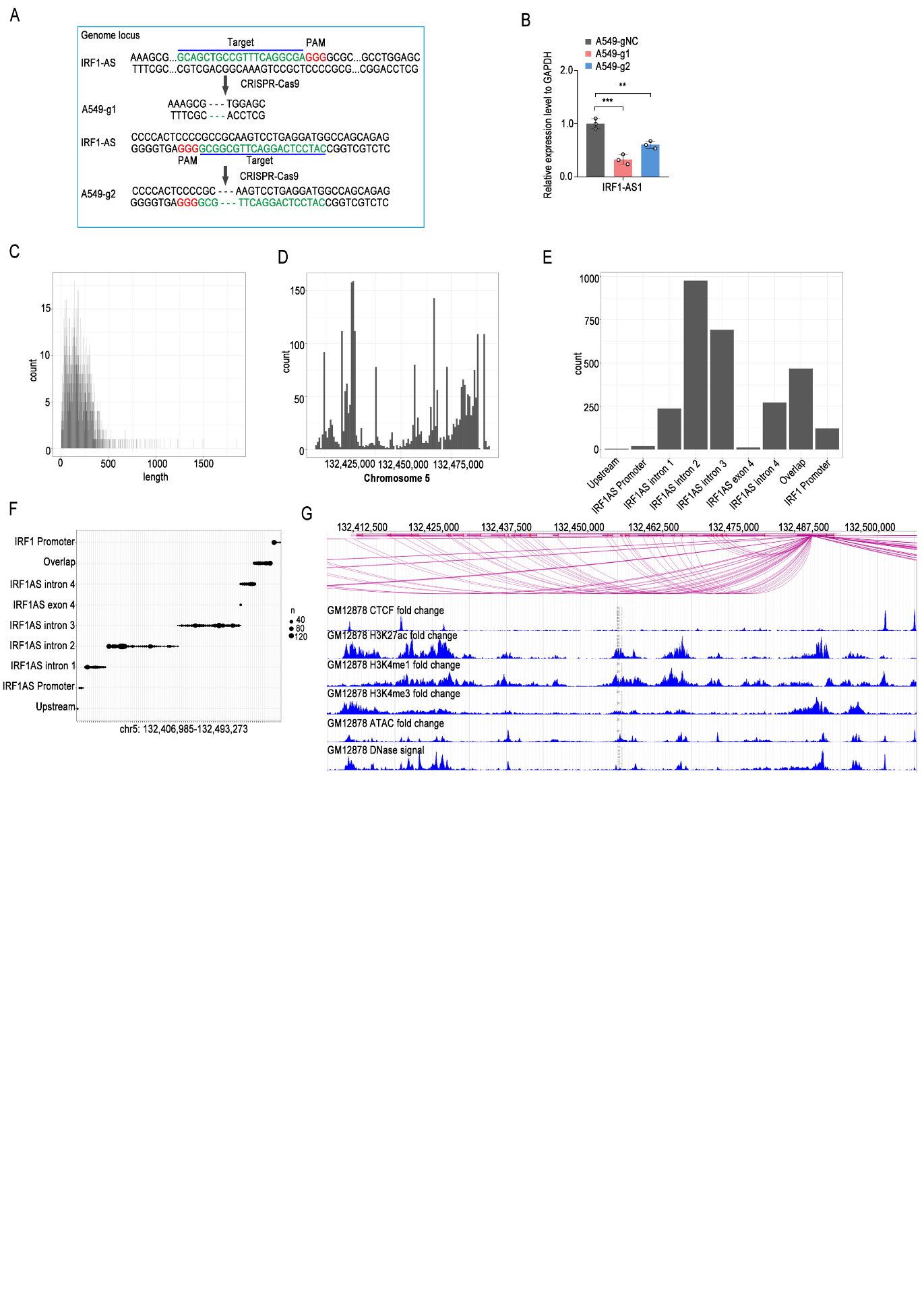


**Fig S5. Characterization and distribution analysis of eRNAs between the IRF1AS and IRF1 gene loci.** (A) Corresponding to Figure 5 (A), the schematic diagram illustrates the CRISPR-Cas9 system targeting exon 1 and exon 2 of IRF1AS for knockout. Green bases denote the target sequence, and red bases indicate the PAM sequence. Following puromycin screening, A549-g1 with a 66-base pair deletion and A549-g2 with a 3-base pair deletion were generated. Cell line sequencing data is available in the supplementary material. (B) The RNA levels of lncRNA IRF1-AS1 were evaluated using qPCR in A549-gNC，knockout cells A549-g1 and A549-g2. (C) Original data were retrieved from the online database eRNAbase via a Genomic Region search at chr5:132,406,985–132,493,273 (https://bio.liclab.net/eRNAbase/index.php) (see Table S2 for details). A bar graph illustrates the length distribution of the eRNAs. (D) The IRF1AS and IRF1 gene loci (chr5:132,406,985–132,493,273) were divided into 800-bp intervals, and a histogram was generated to show the number of eRNAs detected in each partition. (E) The IRF1AS and IRF1 loci were subdivided into regions including Upstream, IRF1AS Promoter, IRF1AS Exon 1, IRF1AS Intron 1, IRF1AS Exon 2, IRF1AS Intron 2, IRF1AS Exon 3, IRF1AS Intron 3, IRF1AS Exon 4, IRF1AS Intron 4, Overlapping regions, IRF1 Exons, and the IRF1 Promoter. The distribution of eRNAs across these subregions is shown in a bar graph (see Table S2 for details). (F) The subregions defined in (E) were mapped onto the 800-bp partitions described in (D). A dot plot presents the number of eRNAs detected in each interval. (G) The distal regulatory interaction within the chr5:132406985-132493273 genomes was examined utilizing Delta.EPI (https://ngdc.cncb.ac.cn/deltaEPI/), an online tool for Enhancer-Promoter Interaction (EPI) annotation from publicly accessible 3D genome data. The above analyses and visualizations were performed using the R package ggplot2. Statistical differences between designated groups (B) were assessed by one-way ANOVA followed by Dunnett's multiple comparisons test. **, *P* < 0.01; ***, *P* < 0.001.


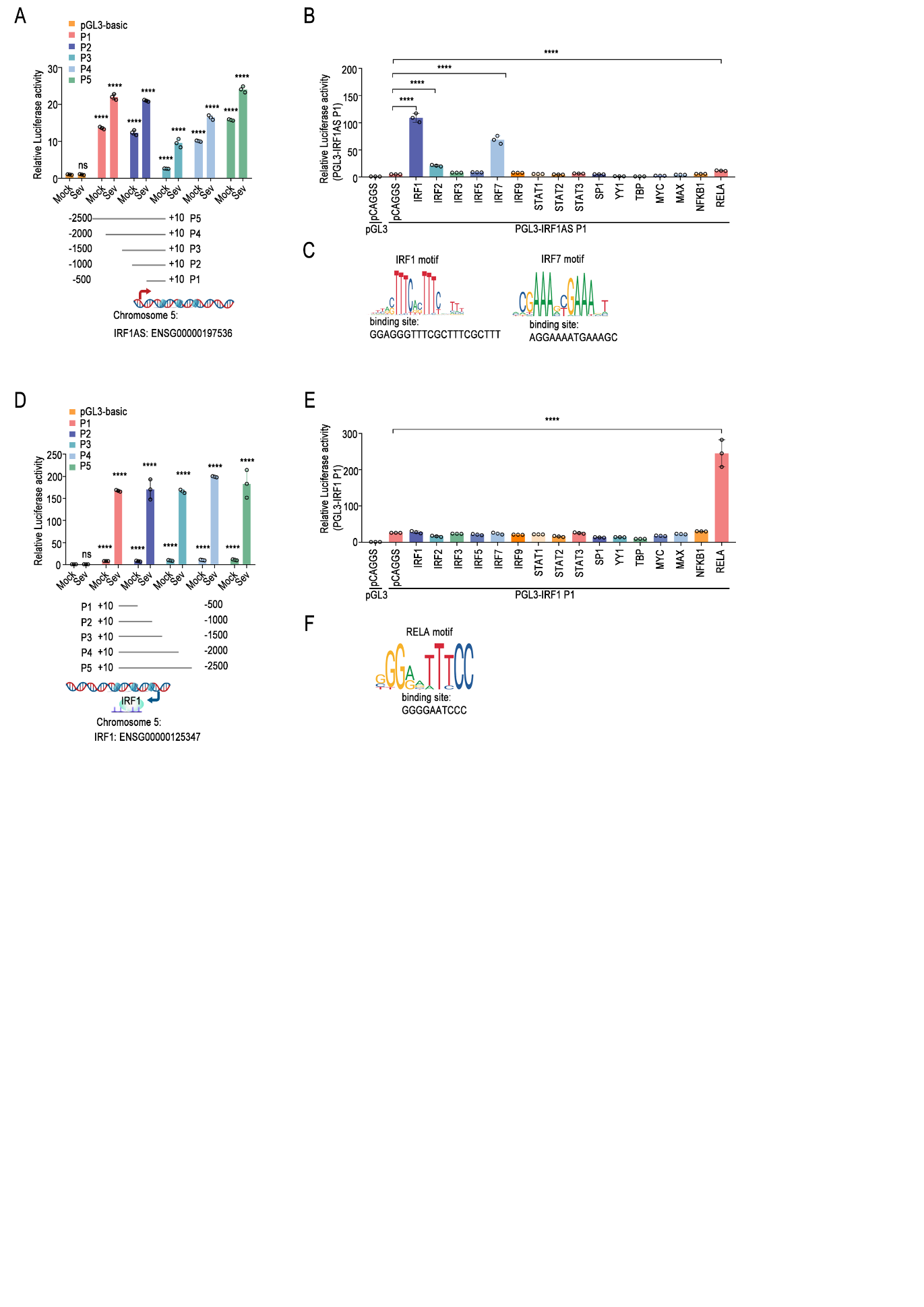


**Fig S6. Screening and identification of promoters and related transcription factors of IRF1AS and IRF1.** (A) Reporter plasmids were constructed by inserting sequences from 10 bp downstream of the IRF1AS transcription start site to 500, 1000, 1500, 2000, and 2500 bp upstream into the pGL3-Basic vector. A luciferase reporter assay was conducted to evaluate promoter activity. (B) The reporter plasmid pGL3-IRF1AS P1 and the pRL-TK plasmid were co-transfected into HEK293T cells along with either the empty pCAGGS vector or expression plasmids encoding specific transcription factors. Promoter activity was assessed using a luciferase reporter assay. (C) Predicted transcription factor binding profiles were generated using the JASPAR online database. (D) A similar experimental design to (A) was applied to the IRF1 gene. (E) A similar approach to (B) was employed for IRF1. (F) A corresponding analysis to (C) was performed for IRF1. The results are presented as means ± standard deviations. Statistical differences among groups were analyzed using one-way ANOVA or two-way ANOVA with Dunnett's multiple comparisons test, using the Mock group (A and D) or the pCAGGS group (B and E) as controls. ****, *P* < 0.0001; ns, no significance.


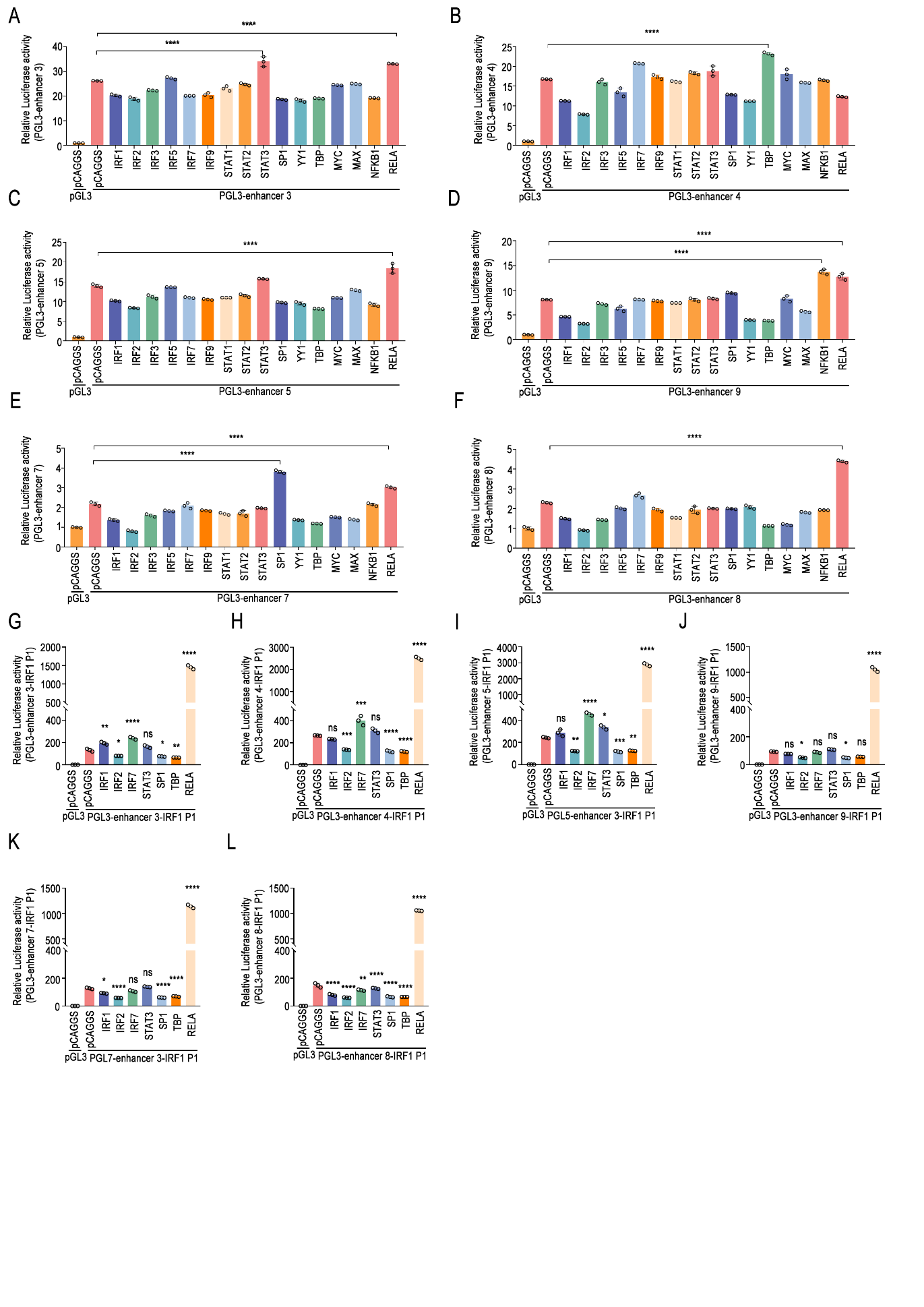


**Fig S7. Identification of the associated transcription factors for each enhancer.** (A–F) The reporter plasmids described in Figure 5D, along with the pRL-TK plasmid and either the empty pCAGGS vector or specified transcription factor expression plasmids, were co-transfected into HEK293T cells. A luciferase reporter assay was performed to assess promoter activity. (G–L) The reporter plasmids described in Figure 5F, together with the pRL-TK plasmid and either specified gene expression plasmids or an empty vector, were co-transfected into HEK293T cells. A luciferase reporter assay was conducted to evaluate the effects of various factors on promoter activity. In panels A-L, statistical differences among groups were shown according to one-way ANOVA with Dunnett's multiple comparisons test, using pCAGGS (empty vectors) as control groups, respectively. *, *P* < 0.05; **, *P* < 0.01; ***, *P* < 0.001; ****, *P* < 0.0001; ns, no significance.

**Table S1. High-throughput sequencing data and microarray datasets used in this study, along with their corresponding annotation information.**

**Table S2. Search results from the eRNAbase database, annotation information for eRNAs, and analysis of the IRF1AS enhancer.**

**Table S3. All primers, siRNAs, oligonucleotides, antibodies, gene sequences, and cell line sequencing data used in this study.**
